# Supplementary material for: ATXN2-mediated translation of TNFR1 promotes esophageal squamous cell carcinoma via m6A-dependent manner
Source: Mol Ther. 2022 Jan 4;30(3):1089–103. doi: 10.1016/j.ymthe.2022.01.006 (PMC8899599; doi:10.1016/j.ymthe.2022.01.006)
Supplement: Document S1. Figures S1–S5 and Tables S2–S4 and S6–S8 [file mmc1.pdf]

YMTHE, Volume *30*

Supplemental Information

ATXN2-mediated translation of TNFR1 promotes esophageal squamous cell carcinoma via m^6A-dependent manner

Rui Li, Lingxing Zeng, Hongzhe Zhao, Junge Deng, Ling Pan, Shaoping Zhang, Guandi Wu, Ying Ye, Jialiang Zhang, Jiachun Su, Yanfen Zheng, Shuang Deng, Ruihong Bai, Lisha Zhuang, Mei Li, Zhixiang Zuo, Dongxin Lin, Jian Zheng, and Xudong Huang

Li et al_Supplementary Figure 1

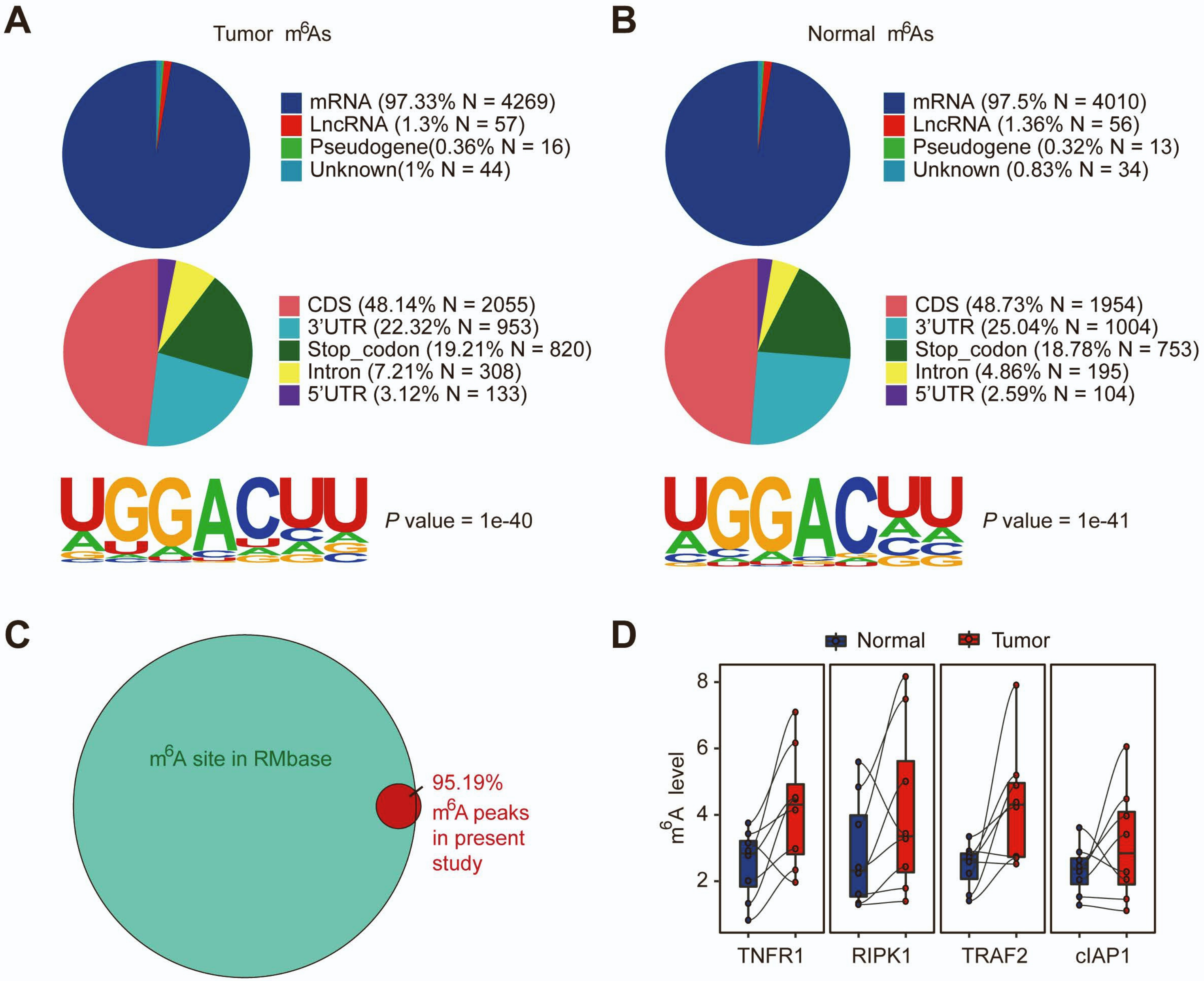

Figure S1. Distribution of RNA m^6A modification in human ESCC. Related to Figure 1.

(A, B) The distribution of annotated m^6A-modified transcripts in different kinds of RNA (*upper panel*: mRNA, lncRNA, pseudogene and others) and a variety of regions (*middle panel*: 5'UTR, CDS, 3'UTR, stop codon and intron). Sequence logo showing the top rank motif detected by Homer (*lower panel*). (C) The Venn plot shows overlap between the m^6A peaks in our m^6A-seq analysis and the m^6A sites in the RMBase database. (D) The m^6A modification level of key molecules (TNFR1, RIPK1, TRAF2 and cIAP1) of TNFR1 signaling in 8 ESCC tumors and paired adjacent normal samples according to m^6A-seq.

Li et al_Supplementary Figure 2

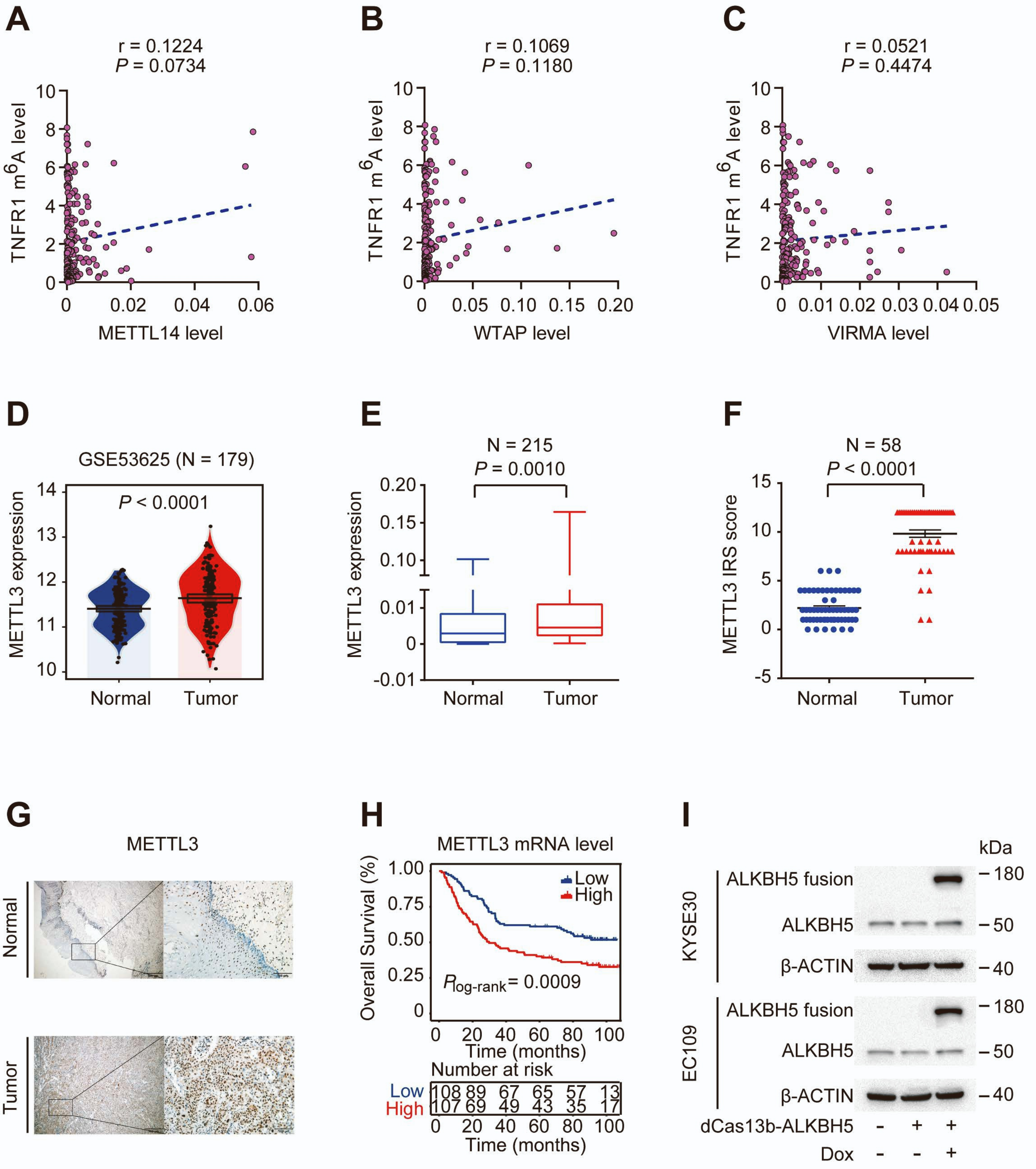

Figure S2. METTL3 expression is significantly upregulated in ESCC. Related to Figure 2.

(A-C) Spearman's correlation analysis between TNFR1 m^6A levels and the mRNA levels of common m^6A writers (METTL14, A; WTAP, B; VIRMA, C) in ESCC tumor tissues (N = 215). (D) METTL3 mRNA expression in ESCC tumors and paired adjacent normal tissues from a public dataset (GSE53625, N = 179). (E) Expression levels of METTL3 RNA in ESCC samples compared with paired normal tissues (SYSUCC cohort, N = 215). (F, G) Quantification of IHC staining (F, SYSUCC cohort, N = 58) and representative IHC images of METTL3 protein levels in ESCC tumors and paired adjacent normal tissues (G). Scale bar, 500 μm (left) and 100 μm (right). (H) Kaplan-Meier estimates of survival time of ESCC patients in SYSUCC Cohort (N = 215) by different METTL3 levels, with the adjusted HRs (95% CI) for death of high METTL3 level being 1.840 (1.284–2.635). (I) Western blot showing the expression of the dCas13b-ALKBH5 fusion protein in ESCC cells with or without doxycycline induction. *P* values were calculated by two-sided paired Wilcoxon signed-rank test in (D), (E) and (F), and two-sided log-rank test was used in (H) ($^*P < 0.05$, $^{**}P < 0.01$ and $^{***}P < 0.001$).

Li et al_Supplementary Figure 3

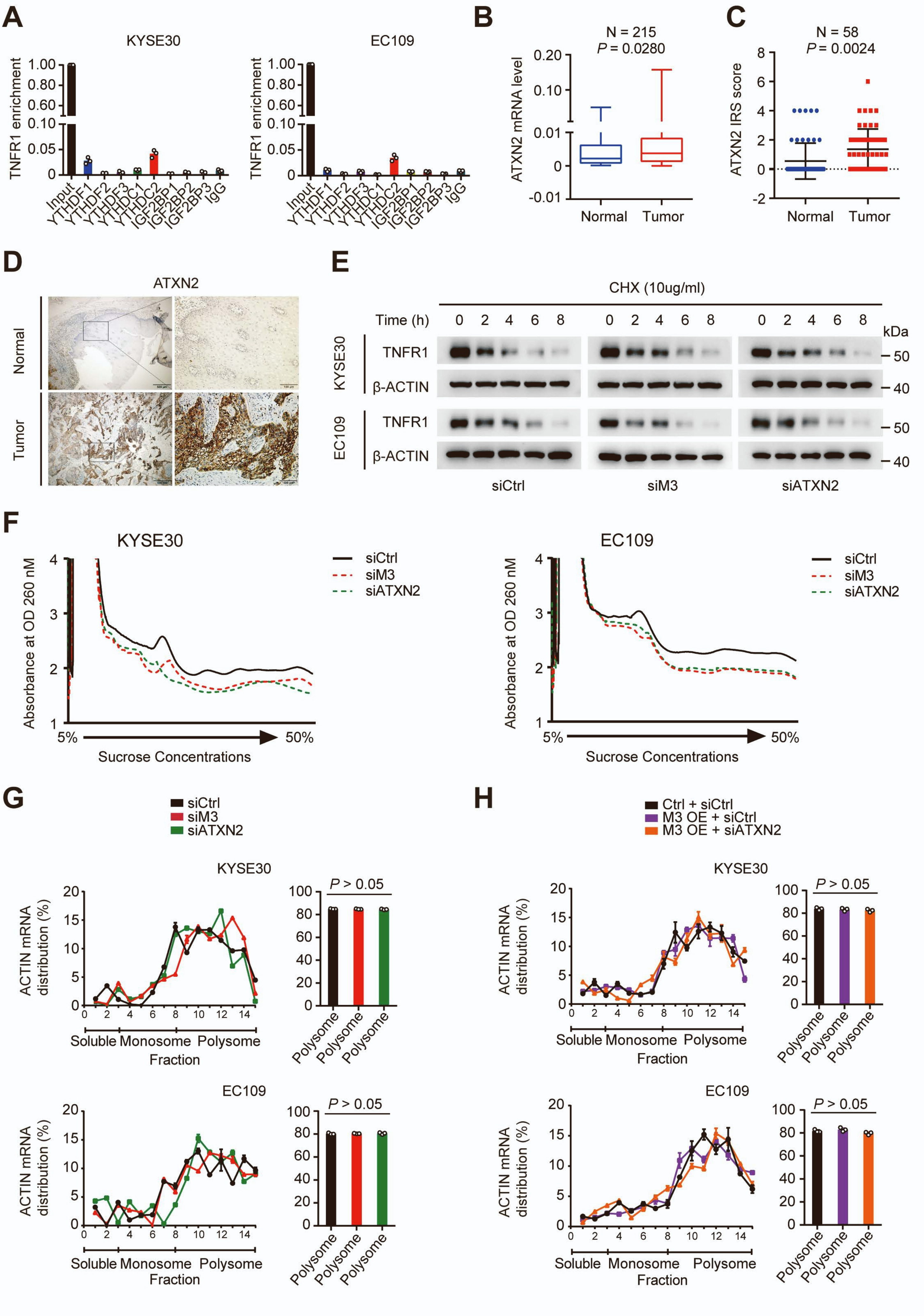

Figure S3. ATXN2 function as a TNFR1 m^{6}A mediator and promotes its translation in ESCC cells. Related to Figure 3.

(A) Association of common readers (YTHDF1/2/3, YTHDC1/2, IGF2BP1/2/3) with TNFR1 determined by RNA immunoprecipitation (RIP) assays. IgG served as the negative control. (B) mRNA levels of ATXN2 in ESCC samples compared with paired normal tissues (SYSUCC cohort, N = 215). (C, D) Quantification of IHC staining (C, SYSUCC cohort, N = 58) and representative IHC images of ATXN2 protein levels in ESCC tumors and paired adjacent normal tissues (D). Scale bar, 500 μm (left) and 100 μm (right). (E) ESCC cells with METTL3/ATXN2 knockdown and control cells were treated with cycloheximide (CHX; 10 μg/ml) for the indicated periods of time. TNFR1 levels were analyzed by immunoblotting. (F) The polysome profiling of ESCC cells with the indicated treatments. (G) Polysome fraction analysis in cells with the indicated treatments. The level of β-ACTIN mRNA in each gradient fraction was measured by qPCR and plotted as a percentage (G, H). The results of (A, G, H) are from at least 3 experiments, and data in (A, G, H) are mean ± SEM. *P*-values were calculated by two-sided Student's *t*-test (**P* < 0.05, ***P* < 0.01 and ****P* < 0.001) in (G, H). *P* values were calculated by two-sided paired Wilcoxon signed-rank test in (B) and (C).

Li et al_Supplementary Figure 4

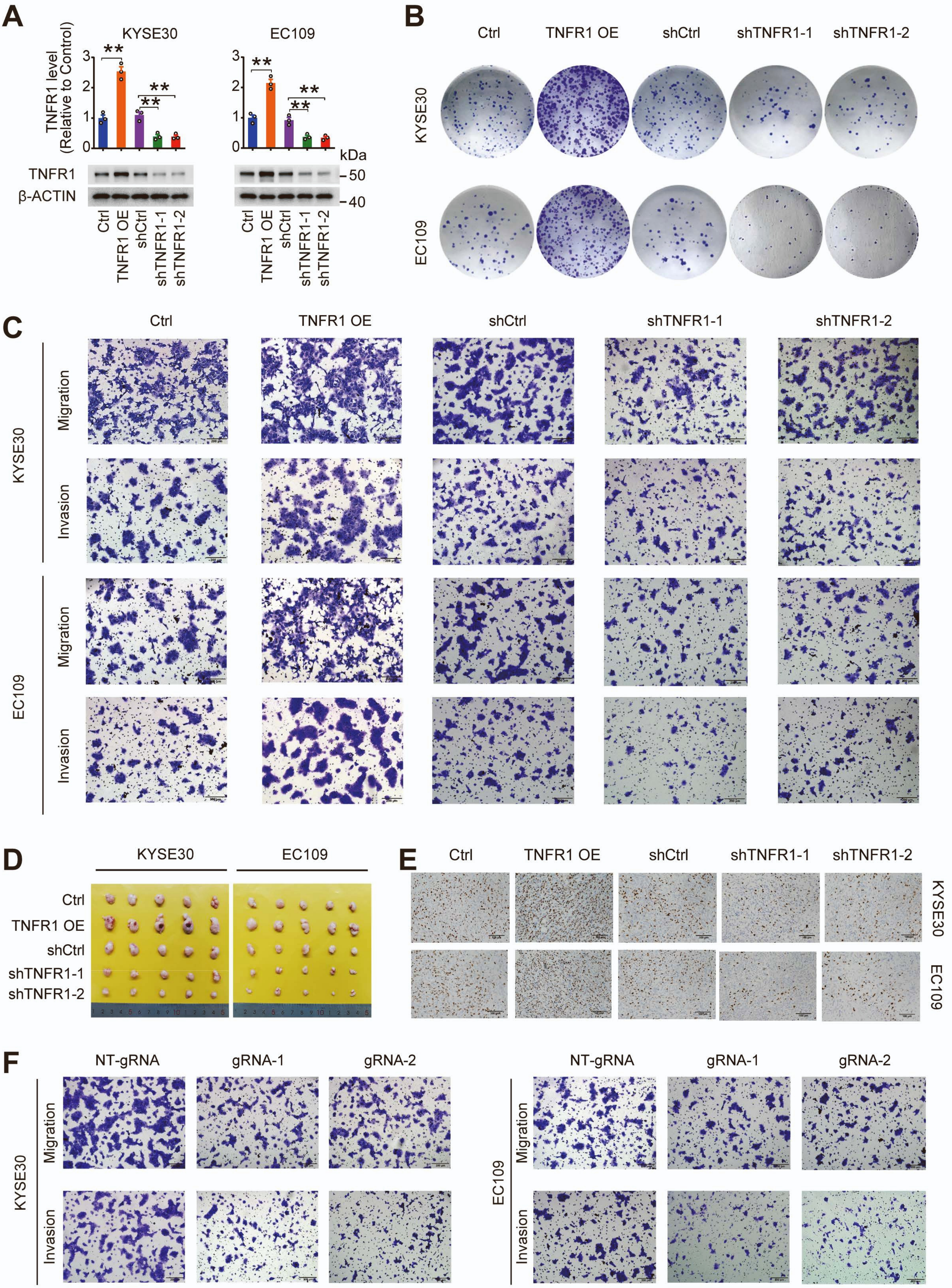

Figure S4. Effects of TNFR1 on ESCC cell malignant phenotypes. Related to Figure 5.

(A) Efficiency of TNFR1 overexpression and knockdown in ESCC cells. Data are the mean ± SEM from 3 independent experiments; **, $P < 0.01$, Student's *t*-test. (B) Colony formation assays were performed to detect the long-term proliferation capacity of KYSE30 and EC109 cells with TNFR1 overexpression or knockdown. (C) Representative images showing the effects of TNFR1 overexpression or knockdown on cell migration and invasion. Scale bars, 200 μm. (D) Effects of TNFR1 overexpression or knockdown on subcutaneous ESCC xenograft growth in mice. Images of xenograft tumors. (E) Representative IHC images of Ki67 levels in xenograft tumors from (D). Scale bars, 100 μm. (F) Representative pictures showing the effects on cell migration and invasion when removing TNFR1 m^6A modifications. Scale bars, 200 μm. The results of (A) are from at least 3 experiments, and the data of (D, E) are from 5 mice. Data in (A) are mean ± SEM. *P* values were calculated by two-sided Student's *t*-test (*$P < 0.05$, **$P < 0.01$ and ***$P < 0.001$) in (A).

Li et al_Supplementary Figure 5

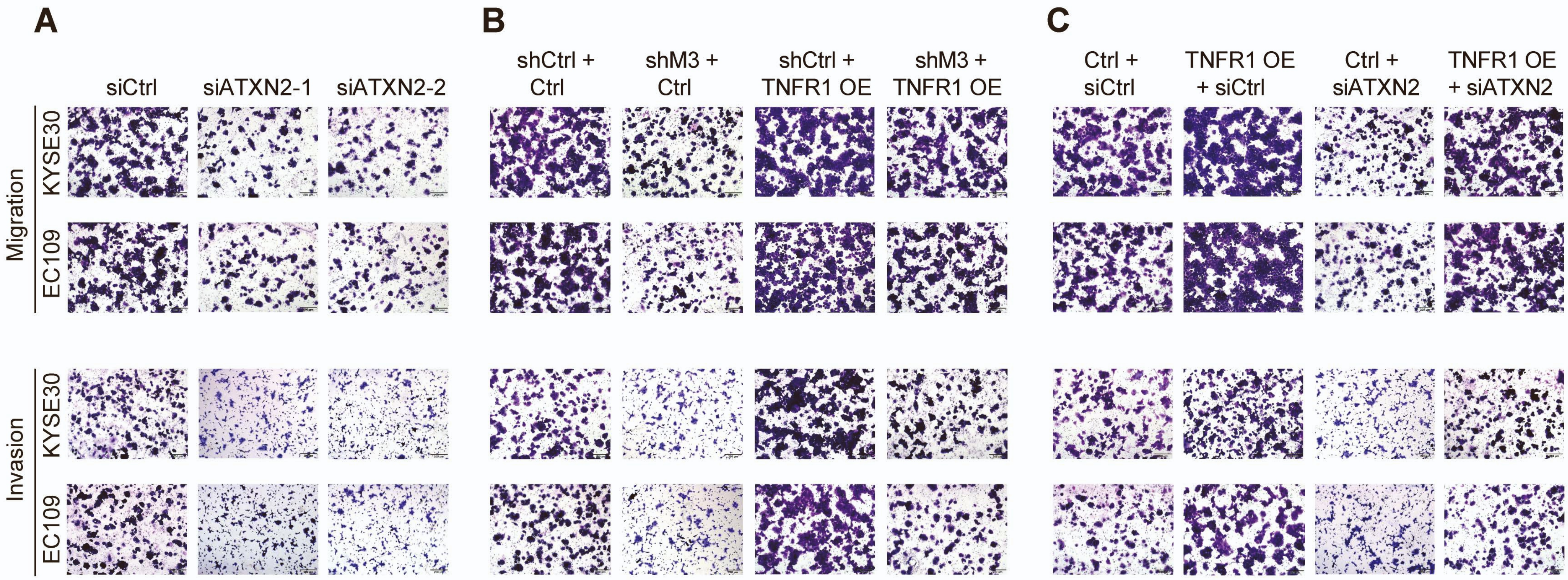

Figure S5. Effects of ATXN2 knockdown and rescue assays on ESCC malignant phenotypes and MAPK/NF-κB activities. Related to Figure 6.

(A) Representative images showing the effects of ATXN2 knockdown on cell migration and invasion. (B) Representative images showing the effects of TNFR1 overexpression on cell migration and invasion in cells with METTL3 knockdown (shM3). (C) Representative images showing the effects of ATXN2 depletion on cell migration and invasion in cells with TNFR1 overexpression (TNFR1 OE). Scale bars, 200 μm in (A−C).

Table S1. Genes involved in TNFR1 related pathways and their m^{6}A levels in ESCC.

Table S2. Baseline and clinical characteristics of patients with ESCC in this study.

**Variable**	**All cases (*N* = 215)**	**Alive (*N* = 93)**	**Deceased (*N* = 122)**	***P* value**[d]
Age, mean (S.E.M.[a])	60.09 (0.62)	59.86 (0.88)	60.27 (0.87)	0.744
Sex, *N* (%)				0.115
Male	166 (77.2)	67 (72.0)	99 (81.1)	
Female	49 (22.8)	26 (28.0)	23 (18.9)	
Family history, *N* (%)				0.631
Yes	52 (24.2)	21 (22.6)	31 (25.4)	
No	163 (75.8)	72 (77.4)	91 (74.6)	
Smoking status[b], *N* (%)				0.351
Ever	137 (63.7)	56 (60.2)	81 (66.4)	
Never	78 (36.3)	37 (39.8)	41 (33.6)	
Drinking status[b], *N* (%)				0.460
Ever	94 (43.7)	38 (40.9)	56 (45.9)	
Never	121 (56.3)	55 (59.1)	66 (54.1)	
Differentiation, *N* (%)				0.858
Well	37 (17.2)	17 (18.3)	20 (16.4)	
Moderate	114 (53.0)	50 (53.8)	64 (52.5)	
Poor	64 (29.8)	26 (27.9)	38 (31.1)	
Tumor stage[c], *N* (%)				< 0.0001
I	13 (6.0)	8 (8.6)	5 (4.1)	
II	93 (43.3)	54 (58.1)	39 (32.0)	
III	101 (47.0)	28 (30.1)	73 (59.8)	
IV	8 (3.7)	3 (3.2)	5 (4.1)	
Treatment, *N* (%)				0.283
Surgery Only	203 (94.4)	87 (93.5)	116 (95.1)	
Surgery + Chemotherapy	5 (2.3)	3 (3.2)	2 (1.6)	
Surgery + Radiotherapy	2 (1.0)	2 (2.2)	0 (0.0)	
Surgery + Chemoradiotherapy	5 (2.3)	1 (1.1)	4 (3.3)	

[a)] S.E.M., standard error of mean.

[b)] Patients were defined as nonsmokers who smoke an average of <1 cigarette/day and for <1 year during their lifetime; other people were defined as smokers. Patients were classified as drinkers who drink at least twice a week and continuously for at least 1 year in their lifetime; other people were defined as nondrinkers.

[c)] Tumor stage were defined according to the 7th edition of the AJCC Cancer Staging System.

[d)] *P* value was calculated by two-sided Chi-square test/Fisher's exact test.

Table S3. Baseline and clinical characteristics of patients with ESCC used for IHC in this study.

**Variable**	**All cases (*N* = 58)**	**Alive (*N* = 24)**	**Deceased (*N* = 34)**	***P* value[d]**
Age, mean (S.E.M.[a])	62.24 (1.14)	62.17 (1.44)	62.29 (1.68)	0.957
Sex, *N* (%)				0.313
Male	46 (79.3)	17 (70.8)	29 (85.3)	
Female	12 (20.7)	7 (29.2)	5 (14.7)	
Family history, *N* (%)				1.000
Yes	13 (22.4)	5 (20.8)	8 (23.5)	
No	45 (77.6)	19 (79.2)	26 (76.5)	
Smoking status[b], *N* (%)				0.352
Ever	39 (67.2)	14 (58.3)	25 (73.5)	
Never	19 (32.8)	10 (41.7)	9 (26.5)	
Drinking status[b], *N* (%)				1.000
Ever	26 (44.8)	11 (45.8)	15 (44.1)	
Never	32 (55.2)	13 (54.2)	19 (55.9)	
Differentiation, *N* (%)				0.934
Well	14 (24.1)	6 (25.0)	8 (23.5)	
Moderate	28 (48.3)	12 (50.0)	16 (47.1)	
Poor	16 (27.6)	6 (25.0)	10 (29.4)	
Tumor stage[c], *N* (%)				0.224
I	3 (5.2)	2 (8.3)	1 (2.9)	
II	22 (38.0)	12 (50.0)	10 (29.4)	
III	31 (53.4)	9 (37.5)	22 (64.8)	
IV	2 (3.4)	1 (4.2)	1 (2.9)	

[a)] S.E.M., standard error of mean.

[b)] Patients were defined as nonsmokers who smoke an average of <1 cigarette/day and for <1 year during their lifetime; other people were defined as smokers. Patients were classified as drinkers who drink at least twice a week and continuously for at least 1 year in their lifetime; other people were defined as nondrinkers.

[c)] Tumor stage were defined according to the 7th edition of the AJCC Cancer Staging System.

[d)] *P* value was calculated by two-sided Chi-square test/Fisher's exact test.

Table S4. Proteins identified in RNA pulldown and mass spectrometry analysis with TNFR1[m^{6}A] or TNFR1[A]

Table S5. Characteristics of ESCC patients for m^{6}A-seq in this study.

Sample ID	Gender	Age	Differentiation	Smoking status[a]	Drinking status[b]	Family history	Tumor stage[c]
320069	Male	53	Poor	Smoker	Nondrinker	No	III
318599	Male	53	Moderate	Smoker	Drinker	Yes	III
322954	Male	68	Moderate	Smoker	Drinker	No	III
320754	Male	59	Poor	Smoker	Nondrinker	No	III
349968	Male	61	Moderate	Smoker	Drinker	No	III
345487	Female	65	Poor	Nonsmoker	Nondrinker	No	III
350991	Male	62	Moderate	Nonsmoker	Drinker	Yes	II
335239	Male	53	Moderate	Smoker	Nondrinker	No	III

a) Patients were defined as nonsmokers who smoke an average of <1 cigarette/day and for <1 year during their lifetime; other people were defined as smokers.

b) Patients were classified as drinkers who drink at least twice a week and continuously for at least 1 year in their lifetime; other people were defined as nondrinkers.

c) Tumor stage were defined according to the 7th edition of the AJCC Cancer Staging System.

Table S6. Primers, oligonucleotides and sgRNAs used in this study.

**qRT-PCR**	Primer sequence (5' → 3')
METTL3-Forward	GTGACTATGGAACCAAGGAGGAG
METTL3-Reverse	TAAGGAAAGAGCAGTCACCTAAAGA
METTL14-Forward	TGCAGCACCTCGATCATTTATTT
METTL14-Reverse	AAGTCTTAGTCTTCCCAGGATTGTT
WTAP-Forward	GCCCAACTGAGATCAACAATGG
WTAP-Reverse	TGGCTATCAGGCGTAAACTTCC
VIRMA-Forward	TCCCAACGATGGCACGAAT
VIRMA-Reverse	TTGCAGCACACCAGGGTGAGC
TNFR1-Forward	AACGGTGGAAGTCCAAGCTCTAC
TNFR1-Reverse	AAGGTGGAACTGGGCACGGGA
ATXN2-Forward	CCTTCAATACTTAGTAACACGGAGCA
ATXN2-Reverse	CATTGGGATTCAATGTTGATTTCCTAA
β-ACTIN-Forward	ACAGAGCCTCGCCTTTGCCGAT
β-ACTIN-Reverse	CTTGCACATGCCGGAGCCGTT
**m^6A-RIP-qPCR**	Primer sequence (5' → 3')
TNFR1-Forward	TGCCTGGACAAGCACATAGCAAG
TNFR1-Reverse	GTGTATGTACAAAAGTCCACAGCTCC
**PAR-CLIP-qPCR**	Primer sequence (5' → 3')
TNFR1-Forward	TGCCTGGACAAGCACATAGCAAG
TNFR1-Reverse	GTGTATGTACAAAAGTCCACAGCTCC
**Select qPCR**	
TNFR1-select up oligonucleotides	tagccagtaccgtagtgcgtgTTGCTATGTGCTTG
TNFR1-select down oligonucleotides	5phos/CCAGGCAGAGGGCACAGGAGcagagg ctgagtcgctgcat
qPCR up primer	ATGCAGCGACTCAGCCTCTG
qPCR down primer	TAGCCAGTACCGTAGTGCGTG
**dCas13b-ALKBH5**	
sgRNA-1	AAAACAAAACAAAACAAAAACAAAAAA AACTGCTTATGCA
sgRNA-2	CTGTGAAAAAGGCTCAGGGACGAACCAG GGGCCCCCGAGC

Table S7. Sequences of shRNAs or siRNAs and probes used in this study**.**

**shRNA or siRNA**	**Sequence (5' → 3')**
shControl	TTCTCCGAACGTGTCACGT
shMETTL3-1	GGAGATCCTAGAGCTATTA
shMETTL3-2	GCACATCCTACTCTTGTAA
shTNFR1-1	GCCATGCAGGTTTCTTTCTAA
shTNFR1-2	CATTGGTTTAATGTATCGCTA
siControl	UUCUCCGAACGUGUCACGUTT
siATXN2-1	GCCCAUGCCAGUGAAUCAATT
siATXN2-2	CCAGCUUACUCCACGCAAUTT
siMETTL3	GGAGAUCCUAGAGCUAUUATT
**Probe for RNA pulldown and EMSA**	**Sequence (5' → 3')**
TNFR1[A]	ACUUGGCACUCCUGUGCCCUCUGCCUGGA[A]CAAGCACAUAGCAAGCUGAAC
TNFR1[m^6A]	ACUUGGCACUCCUGUGCCCUCUGCCUGGA[m^6A]CAAGCACAUAGCAAGCUGAAC

Table S8. Antibodies utilized in this study.

Antibody	Application	Source	Catalog number
Rabbit anti-METTL3	WB, IHC	Abcam	ab195352
Rabbit anti-TNFR1	WB	Cell Signaling Technology	3736
Rabbit anti-Ki67	IHC	ZSGB-BIO	ZA-0502
Rabbit anti-YTHDF1	RIP	Abcam	ab220162
Rabbit anti-YTHDF2	RIP	Abcam	ab220163
Rabbit anti-YTHDF3	RIP	Abcam	ab220161
Rabbit anti-YTHDC1	RIP	Cell Signaling Technology	81504
Rabbit anti-YTHDC2	RIP	Cell Signaling Technology	46324
Rabbit anti-IGF2BP1	RIP	Abcam	ab184305
Mouse anti-IGF2BP2	RIP	Abcam	ab128175
Rabbit anti-IGF2BP3	RIP	Abcam	ab177477
Rabbit anti-ATXN2	WB, RIP, CLIP	Proteintech	21776-1-AP
Rabbit anti-THRAP3	WB, RIP	Invitrogen	A300-956A
Rabbit anti-CCT5	WB, RIP	Invitrogen	PA5-22093
Rabbit anti-MTREX	WB, RIP	Abcam	ab70551
Rabbit anti-p65	WB	Cell Signaling Technology	8242
Rabbit anti-phospho p65	WB	Cell Signaling Technology	3033
Rabbit anti-ERK1/2	WB	Abcam	ab17942
Rabbit anti-phospho ERK1/2	WB	Abcam	ab76299
Rabbit anti-p38	WB	Cell Signaling Technology	8690
Rabbit anti-phospho p38	WB	Cell Signaling Technology	4511
Mouse anti-6-methyladenosine	RIP	Synaptic Systems	202003
Rabbit anti-ALKBH5	WB	Merckmillipore	ABE547
Mouse anti-ACTIN	WB	Proteintech	66009-1-Ig
