# Supplementary material for: ATXN2-mediated translation of TNFR1 promotes esophageal squamous cell carcinoma via m6A-dependent manner
Source: Mol Ther. 2022 Jan 4;30(3):1089–103. doi: 10.1016/j.ymthe.2022.01.006 (PMC8899599; doi:10.1016/j.ymthe.2022.01.006)
Supplement: Document S2. Article plus supplemental information [file mmc4.pdf]

Molecular Therapy
Original Article

ATXN2-mediated translation of TNFR1 promotes esophageal squamous cell carcinoma via m^{6}A-dependent manner

Rui Li,[1,4] Lingxing Zeng,[1,4] Hongzhe Zhao,[1,4] Junge Deng,[1,4] Ling Pan,[1] Shaoping Zhang,[1] Guandi Wu,[1] Ying Ye,[1] Jialiang Zhang,[1] Jiachun Su,[1] Yanfen Zheng,[1] Shuang Deng,[1] Ruihong Bai,[1] Lisha Zhuang,[1] Mei Li,[2] Zhixiang Zuo,[1] Dongxin Lin,[1,3] Jian Zheng,[1] and Xudong Huang[1]

[1]Sun Yat-sen University Cancer Center, State Key Laboratory of Oncology in South China and Collaborative Innovation Center for Cancer Medicine, 651 Dongfeng East Road, Guangzhou 510060, China; [2]Department of Pathology, Sun Yat-sen University Cancer Center, Guangzhou 510060, China; [3]Department of Etiology and Carcinogenesis, National Cancer Center/National Clinical Research Center/Cancer Hospital, Chinese Academy of Medical Sciences and Peking Union Medical College, Beijing 100021, China

N^6-methyladenosine (m^{6}A) is the most prevalent RNA modification, and the effect of its dysregulation on esophageal squamous cell carcinoma (ESCC) development remains unclear. Here, by performing transcriptome-wide m^{6}A sequencing in 16 ESCC tissue samples, we identified the key roles of m^{6}A in *TNFRSF1A* (also known as TNFR1)-mediated MAPK and NF-κB activation in ESCC. Mechanistically, a functional protein involved in m^{6}A methylation, ATXN2, is identified that augments the translation of *TNFRSF1A* by binding to m^{6}A-modified *TNFRSF1A* mRNA. Upregulation of the *TNFRSF1A* protein level, a vital upstream switch for *TNFRSF1A*-mediated signaling events, activates the NF-κB and MAPK pathways and thus promotes ESCC development. Furthermore, *TNFRSF1A* m^{6}A modifications and protein levels are upregulated in ESCC, and high levels of *TNFRSF1A* m^{6}A and protein are correlated with poor ESCC patient survival. These results collectively indicate that the m^{6}A-*TNFRSF1A* axis is critical for ESCC development and thus may serve as a potential druggable target.

INTRODUCTION

Esophageal cancer is the sixth leading cause of cancer-related death worldwide,[1] and the incidence and histological type of esophageal cancer varies with geographic location. Esophageal squamous cell carcinoma (ESCC) is the predominant histological subtype, with poor prognosis and an extremely high prevalence in China.[2] Comprehensive analysis of exome sequencing in tumor-normal paired ESCC tissues identified 22 significantly mutated driver genes (SMGs) (including *TP53*, *NOTCH1*, *NFE2L2*, *KMT2D*, *CDKN2A*, *ZNF750*, *PIK3CA*, *RB1*, *FAT1*, *EP300*, *FBXW7*, *TGFBR2*, *AJUBA*, *CREBBP*, *FAT2*, *NOTCH3*, *PTCH1*, *KDM6A*, *FAM135B*, *TET2*, *PTEN*, and *ADAM29*), along with other somatic genomic alterations contribute to the development of ESCC,[3] but even so, there is still a lack of effective biomarkers for early detection, and the 5-year survival rate of ESCC patients is < 20%.[4] Discovering the molecular mechanisms underlying ESCC development is necessary for developing effective biomarkers and targets for early diagnosis and clinical treatment.

N^6-methyladenosine (m^{6}A) is the most abundant internal reversible chemical modification in eukaryotic mRNA, and "writer" and "eraser" proteins play key roles in the deposition and removal of m^{6}A methylation.[5] RNA m^{6}A modification can be recognized by a "reader" that influences multiple aspects of RNA fate, such as pre-mRNA processing,[6–9] translation,[10] and stability.[11] Emerging evidence has uncovered that m^{6}A modification exerts either oncogenic or tumor-suppressive effects in different conditions.[12,13] Nevertheless, whether and how aberrant m^{6}A abundance can translate to a pro-tumorigenic signal in ESCC is still not understood.

Tumor necrosis factor (TNF), an inflammatory cytokine, plays an important role in the pathogenesis of many chronic inflammatory diseases and cancer by binding two cell-membrane receptors (TNFR1 and TNFR2).[14] It is well known that TNFR1 (also known as *TNFRSF1A*) is ubiquitously expressed and is the primary receptor mediating a majority of the biological effects of TNF.[15] A previous multiple sclerosis genome-wide association study (GWAS) reported

Received 29 June 2021; accepted 3 January 2022;
https://doi.org/10.1016/j.ymthe.2022.01.006.

[4]These authors contributed equally

Correspondence: Dongxin Lin, PhD, Department of Etiology and Carcinogenesis, National Cancer Center/National Clinical Research Center/Cancer Hospital, Chinese Academy of Medical Sciences and Peking Union Medical College, Beijing 100021, China
E-mail: lindx@sysucc.org.cn
Correspondence: Jian Zheng, PhD, Sun Yat-sen University Cancer Center, State Key Laboratory of Oncology in South China and Collaborative Innovation Center for Cancer Medicine, 651 Dongfeng East Road, Guangzhou 510060, China
E-mail: zhengjian@sysucc.org.cn
Correspondence: Xudong Huang, MD, Sun Yat-sen University Cancer Center, State Key Laboratory of Oncology in South China and Collaborative Innovation Center for Cancer Medicine, 651 Dongfeng East Road, Guangzhou 510060, China
E-mail: huangxd@sysucc.org.cn

This is an open access article under the CC BY license (http://creativecommons.org/licenses/by/4.0/).

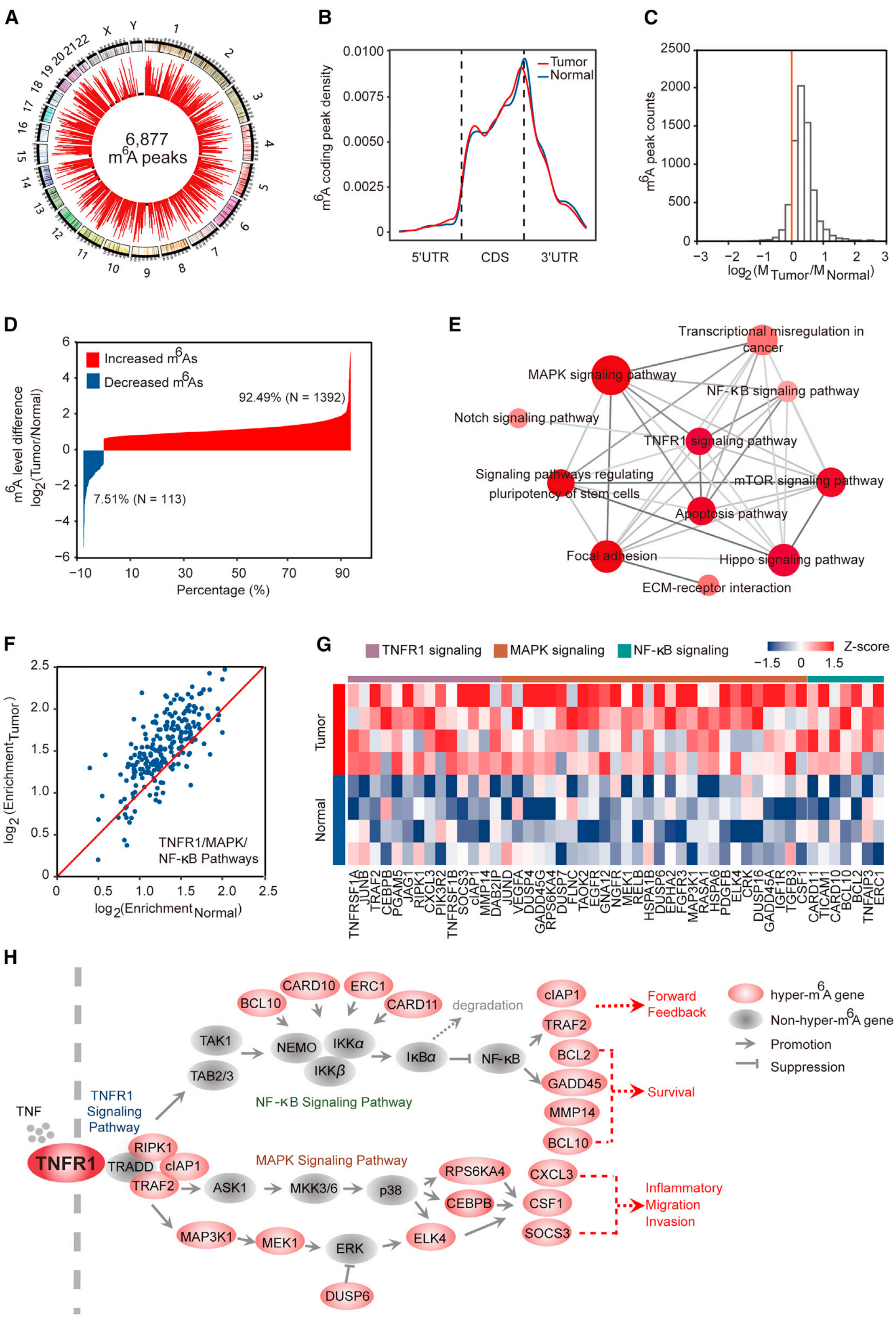

(legend on next page)

that rs1800693 polymorphism, located in the intron of TNFR1, is the most significant signal for multiple sclerosis susceptibility. The risk G allele resulted in the production of a novel, soluble form of TNFR1 that can block TNF. This result indicates that the multiple sclerosis-associated TNFR1 variant simulates the effect of TNF-blocking drugs.[16] TNFR1-mediated signaling has recently been shown to enhance tumor formation during liver,[17] skin,[18] and gastric[19] carcinogenesis and promote the metastasis of cancer cells.[20] Despite that the mechanism of TNFR1-mediated signaling events during tumor promotion has been partially delineated, little is known about ESCC development, especially TNFR1-involved epigenetic regulation.

Here, we revealed an aberrant increase in m^{6}A modification of mRNAs involved in TNFR1-mediated signaling pathways in ESCC tissues. TNFR1 RNA, an upstream switch for TNFR1-mediated signaling events, is aberrantly m^{6}A modified due to the elevated expression of METTL3. An excessive m^{6}A level of TNFR1 promotes protein translation through ATXN2-dependent regulation and evokes mitogen-activated protein kinase (MAPK) and nuclear factor κB (NF-κB) activation, which may be an important molecular mechanism for ESCC development and progression.

RESULTS

TNFR1-involved signaling pathways exhibited elevated m^{6}A modification levels in ESCC

To describe the global landscape of m^{6}A modification in ESCC, we performed m^{6}A sequencing (m^{6}A-seq) of total RNAs of 16 tumor tissues and paired adjacent normal tissues from 8 ESCC patients. We detected 6,877, 4,524, and 4,245 m^{6}A peaks, representing 6,643, 4,386, and 4,113 transcripts in all of the samples, ESCC tumors, and adjacent normal tissues, respectively (Figures 1A, S1A, and S1B). These m^{6}A modifications occurred mainly in mRNA and were predominantly enriched in coding sequence (CDS) and stop codons, consistent with previous studies[21] (Figures 1B, S1A, and S1B). In addition, the canonical motif of GGm^{6}ACU was also enriched in these detected peaks (Figures S1A and S1B). Among the 6,877 m^{6}A peaks, 6,546 (95.19%) were recorded in the RMBase: http://mirlab.sysu.edu.cn/rmbase/,[22] indicating that the m^{6}A-seq data are reliable (Figure S1C). Interestingly, we found that m^{6}A mRNA methylation was increased globally in ESCC tissues compared with adjacent normal tissues (Figure 1C). Among the dysregulated m^{6}A peaks, the number of peaks exhibiting increased m^{6}A levels in at least 4 paired tumor-normal samples was 1,392 (92.49%), but only 113 peaks (7.51%) showed decreased m^{6}A levels in at least 4 paired tumor-normal samples (Figure 1D).

The enrichment theory was adapted to transcripts exhibiting increased m^{6}A levels in at least 4 paired tumor-normal samples, and the results indicated that the transcripts participating in the TNFR1 pathway and its mediated NF-κB and MAPK signaling were perturbed by increased m^{6}A modification (Figure 1E). The binding of TNF to TNFR1 triggers a series of intracellular events that ultimately result in the activation of NF-κB and MAPK,[23,24] which promotes tumor formation in some types of cancers. Therefore, we hypothesized that increased m^{6}A methylation may promote ESCC development mainly through initiating TNFR1-mediated MAPK and NF-κB activation. Interestingly, most transcripts involved in TNFR1-mediated signaling events showed elevated m^{6}A modification in tumor tissues versus adjacent normal tissues (Figures 1F–1H and S1D; Table S1). These results suggested that an aberrant increase in global m^{6}A modification in ESCC may play an oncogenic role via canonical oncogenic pathways, especially the TNFR1-mediated MAPK and NF-κB pathways.

Elevated m^{6}A modification of TNFR1 RNA augments its protein level in ESCC

As mentioned above, the transmembrane receptor TNFR1 is of critical importance and acts as a trigger for TNFR1-mediated oncogenic pathways. Transcripts encoding TNFR1 showed increased m^{6}A modification levels in ESCC tumors based on m^{6}A-seq (Figure 2A). Then, we detected the m^{6}A levels of the TNFR1 transcript in 215 pairs of ESCCs and adjacent normal esophageal samples (SYSUCC cohort; Table S2) by using methylated RNA immunoprecipitation sequencing (MeRIP)-coupled quantitative real-time-PCR (qRT-PCR) and found that its m^{6}A modification was elevated in ESCC tumors (Figure 2B). Next, we attempted to determine which component in m^{6}A methyltransferase complex participated in m^{6}A modification on TNFR1 transcripts. We found that the mRNA levels of METTL14, WTAP, and VIRMA

Figure 1. TNFR1-involved signaling pathways exhibited elevated m^{6}A modification in ESCC

(A) Circos plots showing a total of 6,877 m^{6}A peaks identified from 8 pairs of ESCC tumors and adjacent normal tissues. Each red bar represents an m^{6}A peak, and the height of the bar indicates the sample numbers supporting this peak. The black background circle represents 2 samples, which is the minimum sample number supporting these high-confidence m^{6}A peaks. (B) Metagene plots showing the distribution of m^{6}A peaks in mRNAs (tumor, 4,269 m^{6}A peaks in mRNAs; normal, 4,010 m^{6}A peaks in mRNAs). (C) Histogram showing the changes in m^{6}A enrichment between normal and tumor samples at all of the peaks. The enrichment values are the mean of ESCC samples or paired normal samples and enrichment in tumor tissue. (D) Bar plot showing the distribution of the different methylation levels of dysregulated m^{6}A peaks (tumor relative to normal). For each pair of samples, we calculated the m^{6}A enrichment changes of each m^{6}A peak in the tumor versus normal and identified the set of dysregulated m^{6}A peaks showing a $\geq$1.5-fold increase/decrease in enrichment in at least 4 pairs of ESCC samples. (E) KEGG pathway analysis of transcripts with elevated m^{6}A levels in tumor tissues versus adjacent normal tissues. The size of the dot represents the number of transcripts with increased m^{6}A modification in the corresponding pathway, and the color of the dot indicates the p value of the pathway. The deeper the red, the smaller the p value. (F) Scatterplot showing the m^{6}A enrichment in ESCC tumor and adjacent normal tissue for m^{6}A-modified transcripts involved in the TNFR1/MAPK/NF-κB pathways. The red line is the y = x line. The enrichment values are the median of ESCC samples or paired normal samples. (G) Heatmap showing m^{6}A levels of 49 hypermethylated genes involved in TNFR1/MAPK/NF-κB-related pathways in 4 pairs of ESCC tumors and adjacent normal tissues. (H) Diagram of genes involved in the TNFR1/MAPK/NF-κB pathway based on the KEGG pathway mapper. Genes with increased m^{6}A transcripts in ESCC tumors are marked in red circles. For TNFR1 signaling, the binding of TNF to TNFR1 leads to the recruitment of many key effector proteins (e.g., TRAF2, RIPK1, cIAP1) into the receptor-proximal complex and initiates downstream events leading to NF-κB activation and MAPK activation.

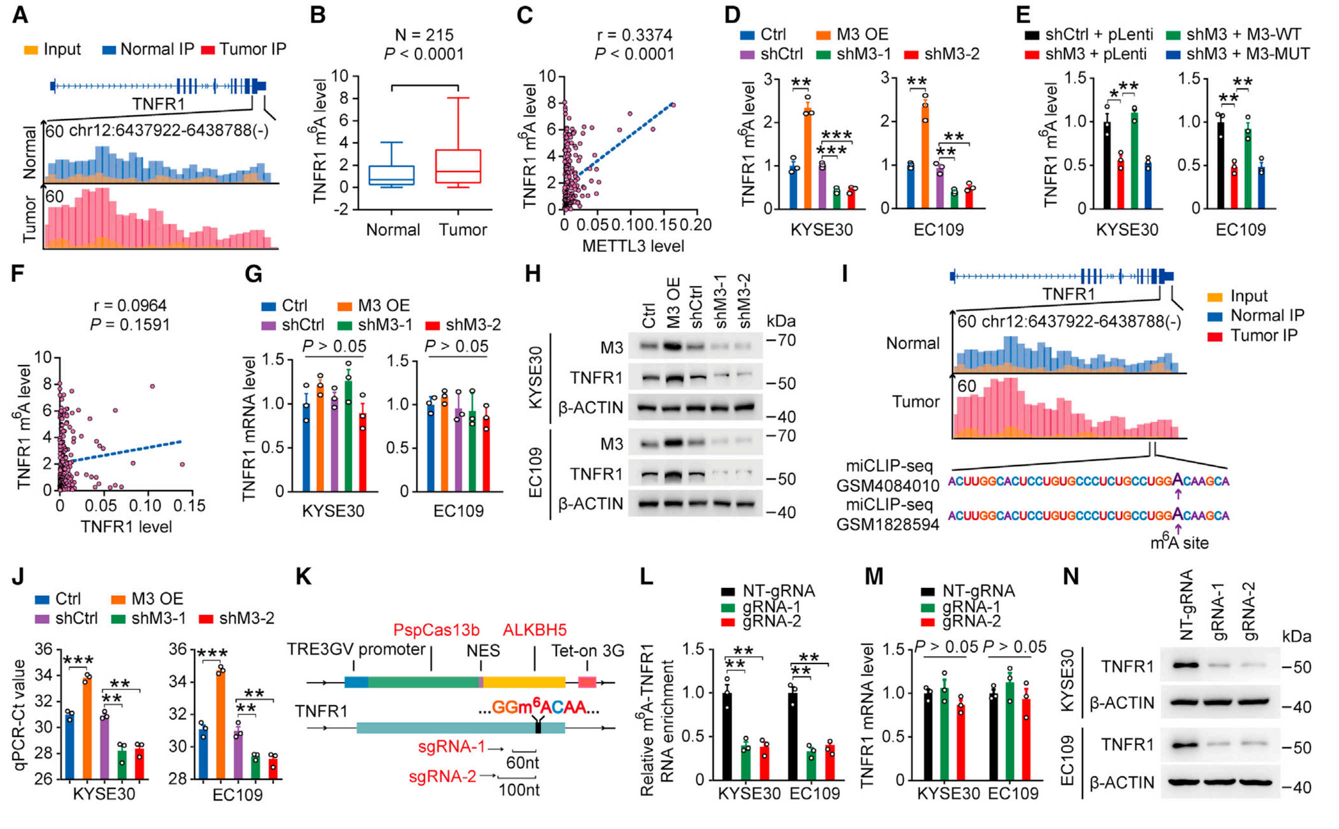

Figure 2. Elevated m^{6}A modification of TNFR1 RNA augments its protein level in ESCC

(A) The average read density from m^{6}A-seq analysis of 8 tumor-normal pairs showing the m^{6}A peak identified in the TNFR1 transcript. (B) TNFR1 m^{6}A levels were significantly higher in ESCC tumors than in paired normal tissues (N = 215). Wilcoxon rank-sum tests (two-sided) were used. (C) Spearman's correlation analysis between TNFR1 m^{6}A levels and METTL3 mRNA levels in ESCC (N = 215). (D) Effects of METTL3 overexpression or knockdown on TNFR1 m^{6}A levels in KYSE30 and EC109 cell lines. (E) Effects of wild-type (M3-WT) or catalytic mutant METTL3 (M3-MUT, aa395-398, DPPW→APPA) overexpression on TNFR1 m^{6}A levels in cells with METTL3 knockdown (shM3). (F) Spearman's correlation analysis between m^{6}A levels and mRNA levels of TNFR1 in ESCC (N = 215). (G) Effects of METTL3 overexpression or knockdown on TNFR1 mRNA levels in KYSE30 and EC109 cell lines. (H) Effects of METTL3 overexpression or knockdown on TNFR1 protein levels in KYSE30 and EC109 cell lines. (I) The average reads density in the m^{6}A peak region of the TNFR1 transcript based on m^{6}A-seq. The sequences around the exact m^{6}A site are highlighted, and the purple arrow indicates the exact m^{6}A site according to 2 public miCLIP-seq datasets (GEO: GSM4084010 and GSM1828594). (J) The TNFR1 m^{6}A levels were measured by the SELECT method in METTL3 overexpression or knockdown cell lines. (K) The schematic represents the domain organization of the dCas13b-ALKBH5 expression cassette (upper panel) and the positions of the m^{6}A site within TNFR1 RNA and regions targeted by 2 gRNAs (lower panel). (L) m^{6}A-TNFR1 RNA levels in ESCC cells treated with doxycycline (DOX)-inducible dCas13b-ALKBH5 plasmid and non-targeting (NT)-gRNA (control) or gRNAs with DOX pretreatment. (M and N) The TNFR1 m^{6}A and protein levels were measured by using the dm^{6}ACRISPR system. Data are the means ± SEMs from at least 3 independent experiments in (D), (E), (G), (J), (L), and (M). Two-sided Student's t tests were used in , (D), (E), (G), (J), (L), and (M) (*$p < 0.05$, **$p < 0.01$, ***$p < 0.001$, and $p > 0.05$, not significant). β-Actin served as loading control in (H) and (N).

were not associated with TNFR1 m^{6}A levels in ESCC tumor tissues (N = 215, SYSUCC cohort; Figures S2A–S2C). However, the m^{6}A levels of the TNFR1 transcript were positively correlated with METTL3 mRNA levels in ESCC tumors (N = 215, SYSUCC cohort; Figure 2C), indicating that METTL3 is the main methyltransferase that catalyzes the m^{6}A modification of the TNFR1 transcript. Meanwhile, we analyzed the expression of METTL3 from three ESCC datasets. METTL3 mRNA expression was significantly upregulated in ESCC tissues compared with adjacent normal tissues in an independent cohort (GSE53625)[25] ($p < 0.0001$, two-sided paired Wilcoxon signed-rank test; Figure S2D). These results were confirmed in our cohort consisting of 215 pairs of ESCC and adjacent normal esophageal samples (SYSUCC cohort; Table S2) by using qRT-PCR ($p = 0.0010$, two-sided paired Wilcoxon signed-rank test; Figure S2E). In addition, immunohistochemistry (IHC) analysis with another 58 paired normal esophageal and ESCC samples showed similar results (SYSUCC cohort; Figures S2F and S2G; Table S3). Kaplan-Meier estimation showed that ESCC patients with high METTL3 mRNA levels (greater than or equal to median) had shorter survival times than ESCC patients with low METTL3 mRNA levels (less than median) (Figure S2H). We then explored the role of METTL3 by disrupting its expression in ESCC cell lines. The overexpression of METTL3 significantly increased the m^{6}A levels of the TNFR1 transcript in two ESCC cell lines, while silencing METTL3 had the opposite effect (Figure 2D). Furthermore, in ESCC cells with METTL3 silencing, the overexpression of wild-type METTL3 (M3-WT), but not the

enzyme-inactivated mutant METTL3 (M3-MUT, amino acids [aa] 395-398, DPPW-APPA[26]), restored the m^{6}A levels in the TNFR1 transcript (Figure 2E), suggesting that m^{6}A modification of the TNFR1 transcript is dependent on the methyltransferase activity of METTL3. We next attempted to determine the function of m^{6}A modification on the TNFR1 transcript. We found that the m^{6}A levels of TNFR1 were not associated with its mRNA abundance (N = 215, SYSUCC cohort; Figure 2F). Moreover, we found that forced changes in METTL3 levels in KYSE30 and EC109 cells did not significantly alter TNFR1 mRNA levels (Figure 2G). However, we observed that upregulation of METTL3 expression significantly increased TNFR1 protein levels and decreased TNFR1 protein levels when METTL3 was silenced (Figure 2H). These results suggested that elevated m^{6}A modification of the TNFR1 transcript may affect its protein translation in ESCC.

We next ascertained an identical m^{6}A modification location at the TNFR1 peak region by using the m^{6}A individual-nucleotide resolution cross-linking and immunoprecipitation sequencing datasets (miCLIP-seq) derived from Gene Expression Omnibus (GEO) database: http://www.ncbi.nlm.nih.gov/geo/ (Figure 2I). Furthermore, the m^{6}A site in the 3′ UTR of TNFR1 was verified by a single-base elongation- and ligation-based quantitative PCR amplification method (called SELECT)[27] in KYSE30 and EC109 cells (Figure 2J). The recently discovered CRISPR-Cas13b-based tool could demethylate targeted m^{6}A-modified mRNA,[28] and we applied the dm^{6}ACRISPR system to TNFR1. The mRNA of TNFR1 was targeted by two guide RNAs (gRNAs) at distinct positions, and the expression of the two gRNAs combined with dCas13b-ALKBH5 significantly decreased the m^{6}A levels of the targeted site (Figures 2K, 2L, and S2I). We examined the mRNA and protein levels of TNFR1 and found that the dm^{6}ACRISPR system significantly reduced the protein level of TNFR1 (Figures 2M and 2N). Overall, excessive m^{6}A modification of TNFR1 RNA augments its protein level in ESCC.

ATXN2 promotes TNFR1 protein translation in an m^{6}A-dependent manner

To gain further insights into the mechanism for how m^{6}A methylation affects the function of TNFR1, we examined common m^{6}A recognition proteins, that is, “reader” proteins, which may be involved in m^{6}A-mediated TNFR1 dysregulation. However, RNA immunoprecipitation (RIP) assays with a series of reader proteins, including YTHDF1-3, YTHDC1-2, and IGF2BP1-3 showed that these common m^{6}A recognition proteins could not interact with TNFR1 transcript (Figure S3A), indicating that there may be other functional proteins involved in this process. We then performed mass spectrometry analysis of proteins generated by RNA pulldown using 50-bp unmethylated or m^{6}A-methylated TNFR1 probes and identified four proteins that potentially interact with m^{6}A-methylated TNFR1 (Figure 3A; Table S4). Western blotting and RIP-coupled qRT-PCR analysis indicated that only ATXN2 bound to m^{6}A-methylated TNFR1 (Figures 3B and 3C). RNA electrophoretic mobility shift assays (REMSA) verified that ATXN2 preferentially bound to m^{6}A-methylated TNFR1 but not unmethylated TNFR1 (Figure 3D). We further examined the mRNA and protein levels of ATXN2 in our ESCC cohort. qRT-PCR and IHC analysis of ATXN2 both revealed that the levels were significantly higher in tumors than in adjacent normal tissue (Figures S3B–S3D), suggesting that ATXN2 may play a role in ESCC development. Analysis of public ATXN2 PAR-CLIP (photoactivatable ribonucleoside-enhanced CLIP) data (POSTAR2 database: http://lulab.life.tsinghua.edu.cn/POSTAR/) from HEK293T cells showed that the ATXN2 binding region covered the m^{6}A site of TNFR1 (Figure 3E). Meanwhile, integration analysis revealed that there is a high degree of co-occupancy between the binding region of ATXN2 and m^{6}A residues (Figure 3F). Furthermore, METTL3 overexpression significantly increased the interaction of ATXN2 with TNFR1 mRNA while it was reduced in METTL3 depletion (Figure 3G). As expected, in METTL3-silenced ESCC cells, overexpression of WT METTL3 but not mutant METTL3 restored the interaction of ATXN2 with TNFR1 mRNA (Figure 3H). We also used the dm^{6}ACRISPR system to remove the m^{6}A modification of TNFR1. CLIP coupled with qRT-PCR assays showed that the interaction between ATXN2 and TNFR1 was significantly decreased after removing the m^{6}A modification (Figure 3I). These results suggested that m^{6}A modification is necessary for the interaction between ATXN2 and TNFR1 RNA.

Next, we wanted to characterize the molecular mechanism underlying interaction between ATXN2 and m^{6}A-modified TNFR1 transcript. We found that ATXN2 silencing in ESCC cells markedly suppressed the protein level of TNFR1 without affecting its mRNA levels (Figures 3J and 3K). Further treatment of ESCC cells silencing METTL3 or ATXN2 with the protein synthesis inhibitor cycloheximide resulted in no difference in half-life for TNFR1 compared with control cells (Figure S3E). We then hypothesized that the potential m^{6}A mediator ATXN2 promotes the translation of TNFR1 in an m^{6}A-dependent manner. The polysome profile showed that ATXN2 knockdown decreased the association of the TNFR1 transcript with actively transcribing ribosomes to a similar extent as METTL3 depletion (Figures 3L, S3F, and S3G). In addition, in METTL3-overexpressing cells, ATXN2 knockdown reversed the increased proportion of TNFR1 mRNA distribution in the polysome fraction (Figures 3M and S3H). These results indicated that the m^{6}A mediator ATXN2 promotes the translation of TNFR1 in an m^{6}A-dependent manner.

m^{6}A methylation regulates the activation of MAPK and NF-κB signaling pathways via TNFR1

Accumulating evidence has indicated that dysregulation of TNFR1 expression contributes to tumorigenesis via its downstream MAPK and NF-κB pathways.[29,30] We examined the mRNA level of TNFR1 in our ESCC cohort by using qRT-PCR and found that there was no significant difference in TNFR1 mRNA levels between ESCC tumor and adjacent normal tissues (Figure 4A). We further performed western blotting to measure the protein level of TNFR1 in 10 paired randomly selected ESCC and adjacent normal tissues. These results revealed that the TNFR1 protein level was increased in ESCC tissues compared with adjacent normal tissues (Figure 4B). IHC analysis with another 58 paired normal esophageal and ESCC samples showed consistent results with western blotting analysis (N = 58, SYSUCC

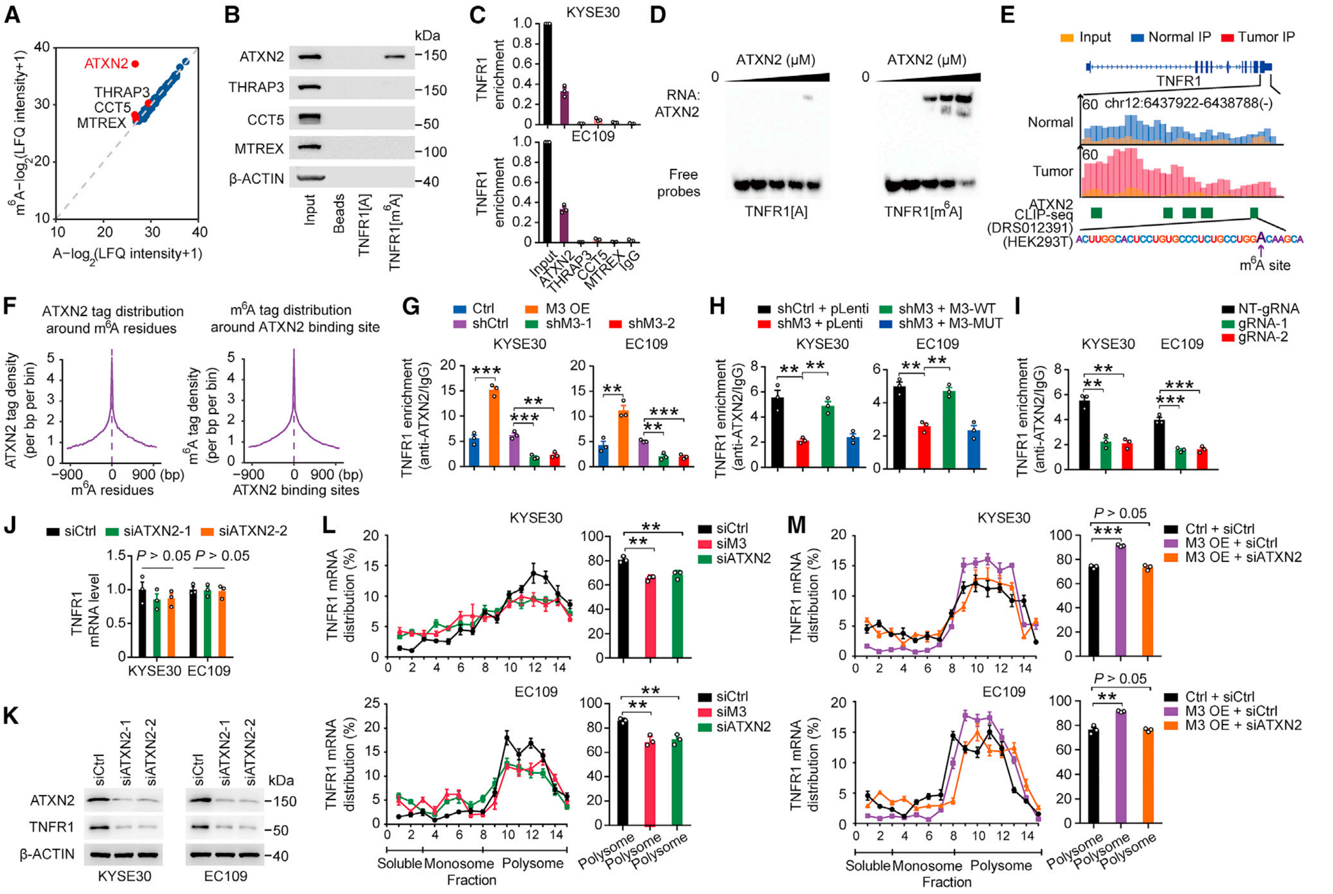

Figure 3. ATXN2 recognizes the TNFR1 m^{6}A site and promotes its translation

(A) Scatterplot of proteins interacting with 50-bp TNFR1 probes with or without m^{6}A modification in KYSE30 cells. The filled red dots indicate potential TNFR1[m^{6}A] binding proteins that have higher affinity for TNFR1[m^{6}A] probes. (B) RNA pull-down coupled with immunoblot analysis shows a specific interaction between ATXN2 and m^{6}A-modified TNFR1. (C) Association of ATXN2 with TNFR1 determined by RNA immunoprecipitation (RIP) assays. IgG served as the negative control. (D) Electrophoretic mobility shift assays of recombinant ATXN2 with unmethylated or methylated TNFR1 probes. The probes were maintained constantly, while a gradient of 0–10 μM recombinant ATXN2 was added to the reactions. (E) Published ATXN2 PAR-CLIP-seq data in HEK293T cells (DRS012391) showed an approximately identical binding region in the TNFR1 transcript with m^{6}A sites. The green bar represents the ATXN2 binding region detected by ATXN2 PAR-CLIP, the sequence of the ATXN2-binding region is highlighted, and the purple arrow indicates the exact m^{6}A site according to 2 public miCLIP-seq datasets. (F) Shown are the intensity of ATXN2 binding centered at m^{6}A residues and the intensity of the m^{6}A CLIP signal centered at ATXN2 binding sites. (G) The levels of ATXN2 bound to TNFR1 RNA determined by CLIP-quantitative PCR in ESCC cells with METTL3 knockdown or overexpression. (H) Effects of WT (M3-WT) or catalytic mutant METTL3 (M3-MUT, aa395–398, DPPW→APPA) overexpression on the association of ATXN2 and TNFR1 in cells with METTL3 knockdown (shM3) determined by ATXN2 CLIP-quantitative PCR. (I) Association of ATXN2 with TNFR1 determined by CLIP-quantitative PCR assays in ESCC cells co-transfected with dCas13b-ALKBH5 and NT-gRNA or gRNAs. (J and K) Effects of ATXN2 knockdown on the mRNA and protein levels of TNFR1 in KYSE30 and EC109 cell lines. (L and M) Polysome fraction analysis in cells with the indicated treatments. The TNFR1 mRNA level in each gradient fraction was measured by quantitative PCR and plotted as a percentage. Data are the means ± SEMs from at least 3 independent experiments in (G)–(J), (L), and (M). Two-sided Student's t tests were used in (G)–(J), (L), and (M) (*$p < 0.05$, **$p < 0.01$, ***$p < 0.001$, and $p > 0.05$, not significant). β-Actin served as loading control in (B) and (K).

cohort; Figures 4C and 4D). We also found that TNFR1 overexpression activated its mediated MAPK and NF-κB pathways in ESCC cells, as indicated by the enhanced phosphorylation of ERK, p38, and p65 without an alteration in their total protein levels, while TNFR1 knockdown exhibited the opposite results (Figure 4E). We next explored whether m^{6}A modification was involved in the activation of TNFR1-mediated signaling pathways. After removing the m^{6}A modification of TNFR1, we found that the phosphorylation of ERK, p38, and p65 protein was significantly decreased compared to that in the control cells, along with TNFR1 protein level reduction (Figure 4F). Furthermore, we investigated the effects of TNF treatment on MAPK and NF-κB activation in ESCC cells by using the dm^{6}ACRISPR system. The results indicated that MAPK and NF-κB signaling were attenuated and persisted for fewer periods of time in ESCC cells when TNFR1 m^{6}A modifications were removed (Figure 4G). These results demonstrated that the upregulation of TNFR1 protein levels caused by elevated m^{6}A modification activates TNFR1-mediated MAPK and NF-κB signaling pathways in ESCC.

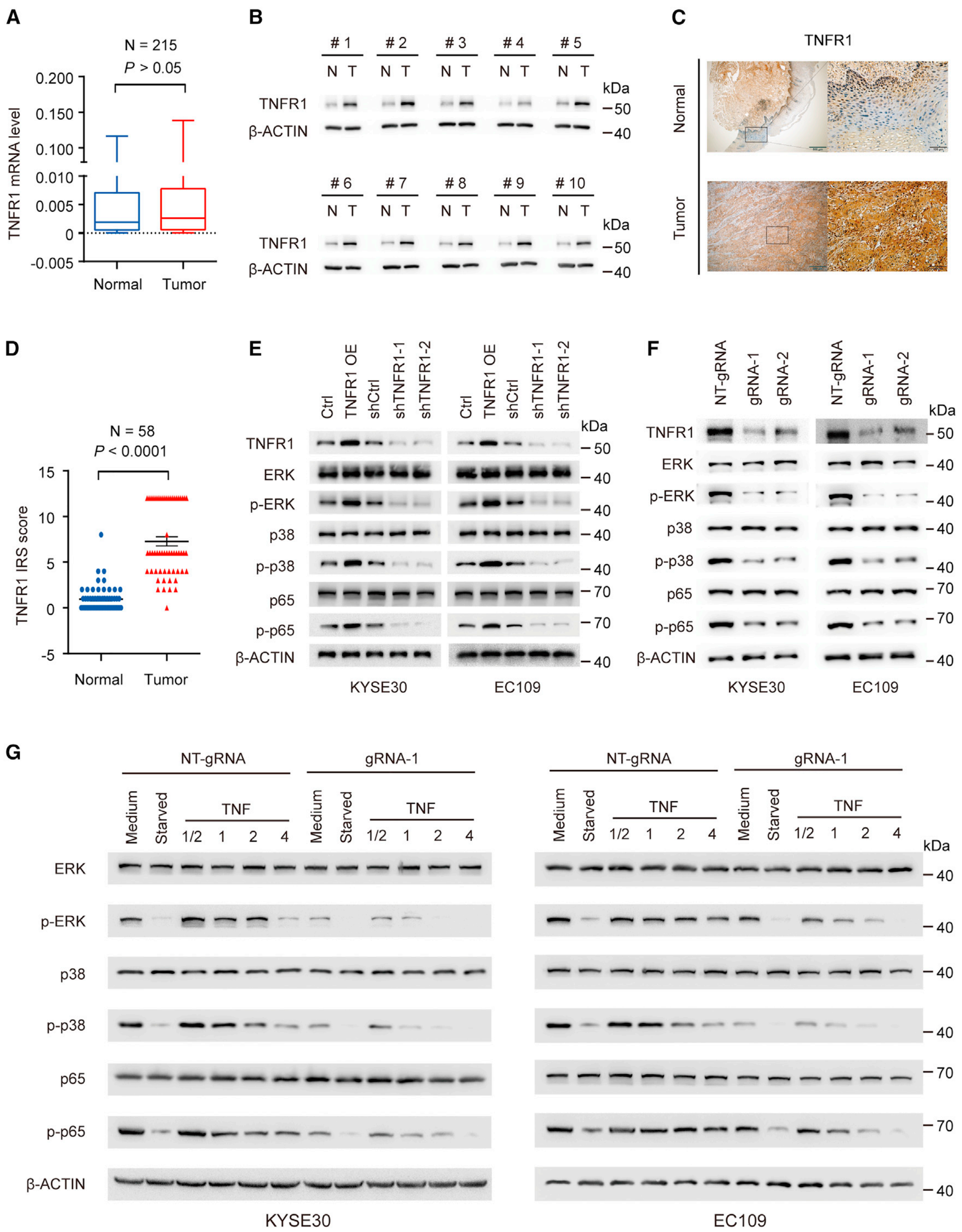

Figure 4. Effects of m^{6}A methylation on TNFR1-mediated MAPK and NF-κB signaling pathway activation

(A) mRNA levels of TNFR1 in ESCC samples compared with paired normal tissues (SYSUCC cohort, N = 215). (B) Immunoblot analysis of the protein levels of TNFR1 in ESCC tissues and corresponding adjacent normal tissues (N = 10). β-Actin was used as a loading control. T, tumor tissues; N, adjacent normal tissues. (C and D) Representative IHC images of TNFR1 in ESCC tumors and paired adjacent normal tissues (C) and quantification of IHC staining (N = 58) (D). Scale bars, 500 and 100 μm. (E and F) Immunoblot assays showed alterations in p-ERK, p-p38, and p-p65 in ESCC cells when TNFR1 was overexpressed or knocked down (E) or when m^{6}A modification was removed by the dm^{6}ACRISPR system (F). (G) Immunoblot assays showed the time course of ERK, p38, and p65 phosphorylation after TNF-α stimulation in ESCC cells treated with the dm^{6}ACRISPR system. p values were calculated by two-sided paired Wilcoxon signed-rank test in (A) and (D) (*p < 0.05, **p < 0.01, ***p < 0.001, and p > 0.05, not significant). β-Actin served as loading control in (B) and (E)–(G).

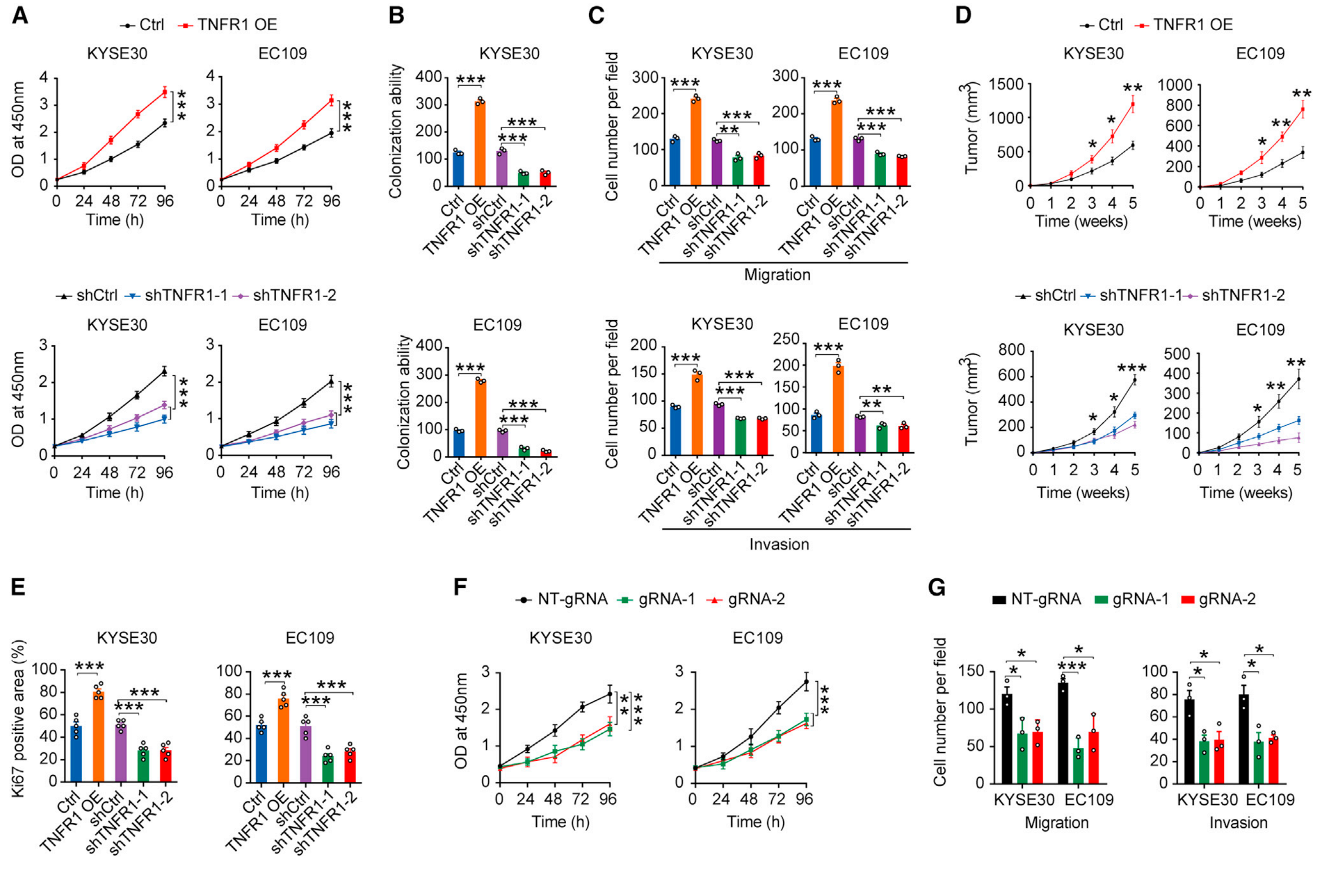

Figure 5. Functional relevance of TNFR1 and its m^{6}A level in ESCC

(A–C) Effects of TNFR1 overexpression or knockdown on ESCC cell proliferation (A), colony formation (B), and migration and invasion (C). (D) Effects of TNFR1 overexpression or knockdown on subcutaneous ESCC xenograft growth in mice. (E) IHC assays determined the effects of TNFR1 overexpression or knockdown on proliferation marker Ki67 in ESCC xenograft (N = 5). (F and G) Effects on ESCC cell proliferation (F) and migration and invasion (G) by using the dm^{6}ACRISPR system. The results of (A), (B), and (F) are from at least 3 experiments; the results of (C) and (G) are from 3 random fields; and the results of (D) and (E) are from 5 mice. Data in (A)–(G) are means ± SEMs. p values were calculated by two-sided Student's t test (*p < 0.05, **p < 0.01, and ***p < 0.001).

The oncogenic roles of METTL3-m^{6}A-TNFR1-ATXN2 axis in ESCC

It has been shown that the TNF receptor TNFR1 plays a key role in tumor development, including skin cancer,[18] gastric cancer,[19] and colon cancer.[20] To examine the biological function of TNFR1 in ESCC, we performed gain- and loss-of-function studies in KYSE30 and EC109 cells. The results revealed that TNFR1 overexpression promoted ESCC cell proliferation, colony formation, migration, and invasion *in vitro*, while TNFR1 knockdown exhibited the opposite effects (Figures 5A–5C and S4A–S4C). In the subcutaneous xenograft tumor model, the tumor volume of the TNFR1 overexpression group was significantly increased compared with that of the control group, while TNFR1 knockdown significantly inhibited tumor growth (Figures 5D and S4D). Consistently, Ki67 IHC assays of xenograft tumor tissues also revealed that TNFR1 overexpression promoted cancer cell proliferation, while TNFR1 knockdown suppressed cancer cell proliferation compared with each control (Figures 5E and S4E). In addition, we observed the suppression of ESCC cell proliferation, migration, and invasion when the m^{6}A modification in TNFR1 was removed (Figures 5F, 5G, and S4F), indicating that the m^{6}A modification of TNFR1 also influences ESCC development.

We also determined the biological function of ATXN2 in ESCC. We found that the depletion of ATXN2 significantly suppressed the proliferation, migration, and invasion of ESCC cells *in vitro* compared to siControl (Figures 6A, 6B, and S5A). Also, knockdown of ATXN2 substantially decreased the phosphorylation levels of ERK, p38, and p65 with no alteration of their total protein levels (Figure 6C). Then, we carried out rescue assays to further verify the METTL3-m^{6}A-TNFR1-ATXN2 axis in ESCC cells. We found that METTL3 depletion inhibited the proliferation, migration, invasion, and phosphorylation levels of ERK, p38, and p65 in ESCC cells. However, the overexpression of TNFR1 in METTL3 depletion cells partially restored the abilities of proliferation, migration, invasion, and phosphorylation levels of ERK, p38, and p65 (Figures 6D–6F and S5B). Similar to Figures 5A and 5C, TNFR1 overexpression increased the

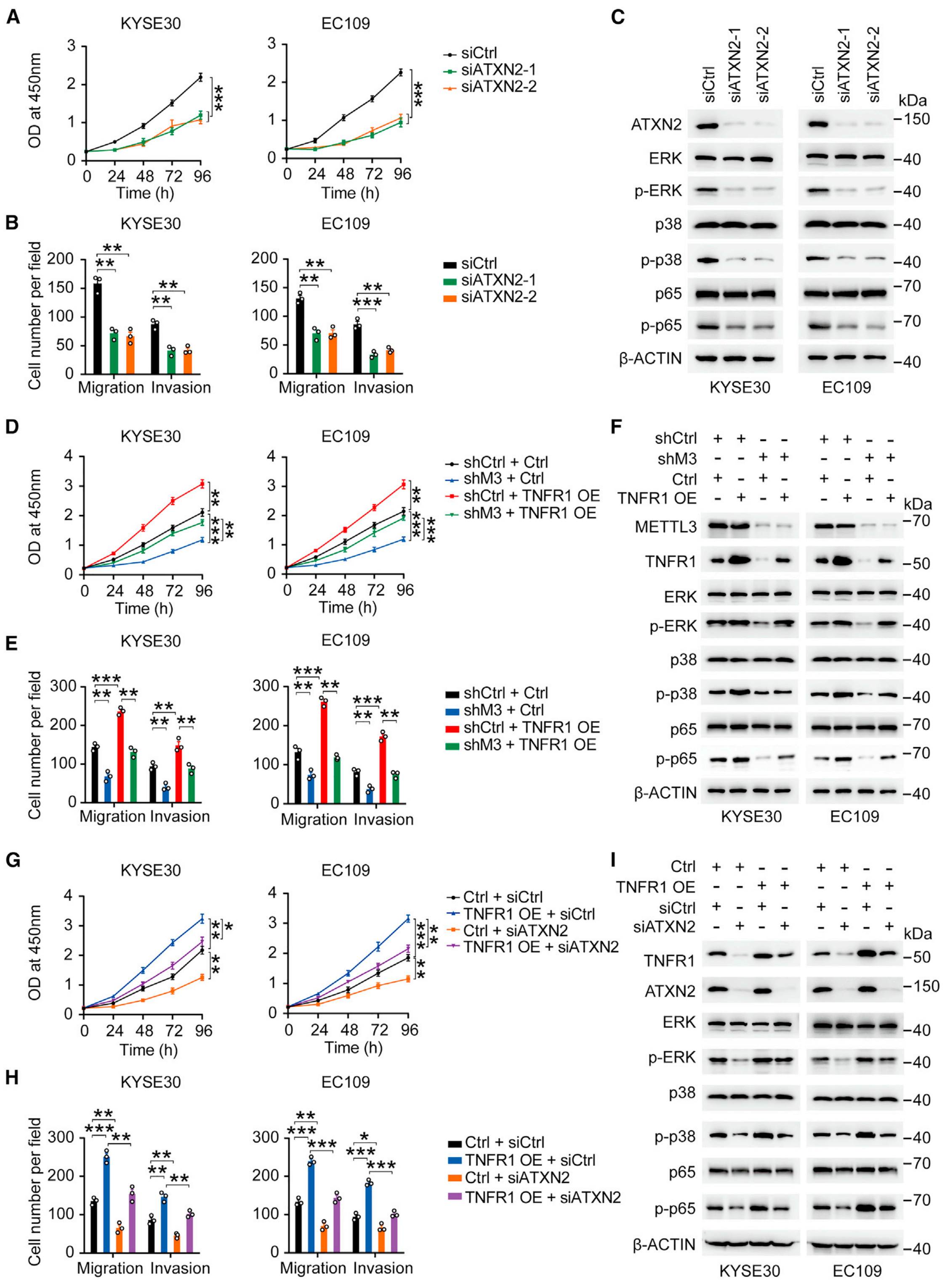

Figure 6. The oncogenic roles of METTL3-TNFR1-ATXN2 axis in ESCC

(A and B) Effects of ATXN2 knockdown on ESCC cell proliferation (A) and migration and invasion (B). (C) Immunoblot assays showed alterations in p-ERK, p-p38, and p-p65 in ESCC cells when ATXN2 was knocked down. (D and E) Effects of TNFR1 overexpression on malignant phenotypes (proliferation, D; migration and invasion, E) in cells with

(legend continued on next page)

proliferation, migration, invasion, and the phosphorylation levels of ERK, p38 and p65 of ESCC cells, but ATXN2 silence in TNFR1 overexpression cells partially repressed the proliferation, migration, invasion, and activation of ERK, p38, and p65 in ESCC cells (Figures 6G–6I and S5C). Collectively, these data suggested that the METTL3-m^{6}A-TNFR1-ATXN2 axis plays oncogenic roles in ESCC through MAPK and NF-κB signaling pathways.

By analyzing our ESCC cohort, we found that the m^{6}A levels of TNFR1 were significantly higher in advanced ESCC stage (III/IV) than in early ESCC stage (I/II) ($p = 0.0018$, Mann-Whitney test; Figure 7A). Kaplan-Meier estimation showed that patients with high m^{6}A levels of TNFR1 (greater than or equal to median) had shorter survival times than patients with low m^{6}A levels (less than median) ($p = 0.0003$, log rank test; Figure 7B). IHC analysis in another 58 paired normal esophageal and ESCC samples (SYSUCC cohort; Table S3) showed that patients with advanced-stage (III/IV) disease were characterized by higher TNFR1 protein levels than those with early-stage disease (I/II) ($p = 0.0013$, Mann-Whitney test; Figure 7C). Survival analysis also indicated that patients with higher TNFR1 protein levels (greater than or equal to median) had shorter overall survival times than patients with low TNFR1 levels (less than median) ($p = 0.0043$, log rank test; Figure 7D). These results reveal that TNFR1 and its m^{6}A level may play an oncogenic role in ESCC development.

DISCUSSION

In the present study, we demonstrated that ESCC undergoes an aberrant increase in global m^{6}A abundance compared with adjacent normal tissues. Among dysregulated m^{6}A sites, 92.49% of m^{6}A sites exhibited increased m^{6}A levels in ESCC; however, 7.51% of m^{6}A sites showed the opposite. Notably, we found that the m^{6}A levels of most mRNAs involved in TNFR1-mediated signaling pathways were elevated in ESCC compared with normal tissues. Since TNFR1 is a critical switch for initiating the above-mentioned signaling events, we focused on the m^{6}A modification of TNFR1 and further investigated its function. Mechanistically, TNFR1 RNA is aberrantly m^{6}A modified due to the elevated expression of METTL3 in ESCC. A m^{6}A mediator, Ataxin-2 (ATXN2), recognizes the m^{6}A site of TNFR1 and promotes protein translation in an m^{6}A-dependent manner. The upregulation of TNFR1 protein expression triggers the activation of MAPK and NF-κB signaling pathways, which may be an important molecular mechanism for ESCC development and progression (Figure 7E).

Here, we generated whole-transcriptome m^{6}A methylomes containing 8 pairs of ESCC tumors and adjacent normal tissues using m^{6}A-seq and verified that increased m^{6}A modification plays an oncogenic role in ESCC. Many studies have reported that TNF mediates the inflammatory response, and continuous activation of TNF signaling has been implicated in the pathogenesis of cancer.[17,31] TNFR1 initiates most of the biological activities of TNF, and the engagement of TNF with its cognate receptor TNFR1 results in the release of the inhibitory protein silencer of death domains (SODDs) and formation of a receptor-proximal complex containing additional adapter proteins (e.g., RIPK1, TRAF2, cIAP1). These latter proteins recruit key enzymes to complexes that are responsible for initiating NF-κB activation and MAPK signaling events.[32,33] TNFR1 is involved in malignant processes through different mechanisms. However, little has been reported on the regulatory mechanism of the response to RNA modification. In this study, we found that TNFR1-involved signaling pathways showed elevated m^{6}A abundance in tumor tissues versus adjacent normal tissues, suggesting that increased m^{6}A modification may play an important role in TNFR1-mediated oncogenic pathways in ESCC.

As is known, reader proteins directly bind and recognize m^{6}A marks on RNA and are responsible for RNA fate (e.g., splicing, export, stability, translation). YTHDF1 and YTHDF3 have been identified as canonical m^{6}A readers that promote target translation.[34,35] In our study, we used a series of assays to identify that ATXN2 is a m^{6}A mediator that recognizes the m^{6}A mark on TNFR1 RNA. A previous study reported that ATXN2 protein, with a molecular weight of 150 kDa, is widely expressed in human tissues.[36] ATXN2 can directly interact with target RNAs via its Lsm and Lsm-AD domains[37] and interact with poly A-binding protein (PABPC1) through its PAM2 motif. The ATXN2-PABPC1 complex plays a role in translation initiation.[38] In amyotrophic lateral sclerosis (ALS), ATXN2 is identified as a dose-sensitive modulator of TDP-43 toxicity; the complex formed by these two proteins is mislocalized in spinal cord neurons and eventually leads to ALS.[39] However, the function of ATXN2 in ESCC development has not been reported yet. Here, we provided strong evidence that ATXN2 promotes the m^{6}A-dependent translation of TNFR1, which may be a potential mechanism of ATXN2-mediated target RNA regulation in ESCC.

Finally, we provided evidence regarding the clinical significance of TNFR1 and its m^{6}A modification in ESCC. Previous studies have reported the oncogenic role of TNFR1 in mouse skin,[18] colon, and gastric tumorigenesis,[19] and our findings consistently demonstrated that TNFR1 promotes ESCC cell proliferation and invasiveness *in vitro*; m^{6}A modification of TNFR1 may play an important role in TNF-induced inflammatory response and tumorigenesis. In addition, we confirmed that the m^{6}A levels and protein levels of TNFR1 were significantly associated with the overall survival rate of ESCC patients, supporting the possibility that TNFR1 acts as a potential therapeutic target in ESCC. Targeting dysfunctional m^{6}A sites by epitranscriptome editing has been developed in recent years as a promising strategy for cancer therapy.[40] Therefore, targeting m^{6}A sites combined with chemotherapy, radiotherapy, or immunotherapy is

METTL3 knockdown (shM3). (F) TNFR1 overexpression partially restored the expression of p-ERK, p-p38, and p-p65 in ESCC cells with shM3. (G–I) Effects of ATXN2 depletion on malignant phenotypes (proliferation, G; migration and invasion, H) and activation of MAPK and NF-κB pathways (I) in cells with TNFR1 overexpression (TNFR1 OE). The results of (A), (D), and (G) are from at least 3 experiments, and the results of (B), (E), and H) are from 3 random fields. Data in (A), (B), (D), (E), (G), and (H) are means ± SEMs. p values were calculated by two-sided Student's t test (*$p < 0.05$, **$p < 0.01$, and ***$p < 0.001$).

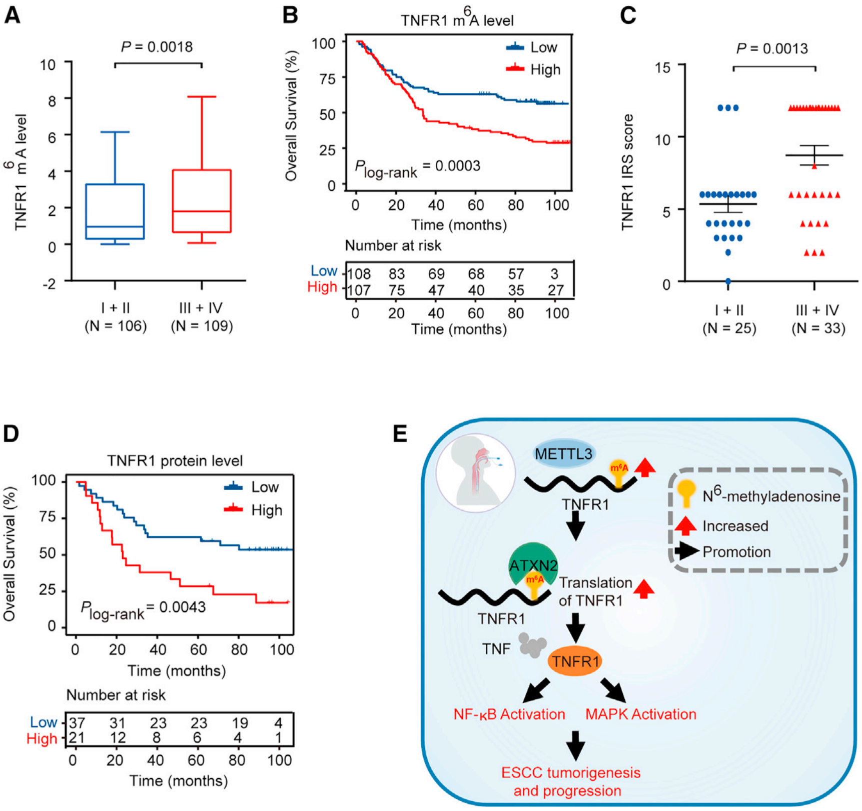

Figure 7. Clinical relevance of TNFR1 and its m^{6}A level in ESCC

(A) TNFR1 m^{6}A levels were significantly higher in stage III/IV ESCC (N = 109) than in stage I/II ESCC (N = 106). (B) Kaplan-Meier estimates of survival time in 2 groups of patients with ESCC and combined samples by different TNFR1 m^{6}A levels in tumors, with a hazard ratio (HR) and 95% confidence interval (CI) of 1.927 (1.349–2.748). (C) Quantification of TNFR1 IHC staining in ESCC tissues showed higher TNFR1 protein levels in stage III/IV ESCC (N = 33) than in stage I/II ESCC (N = 25). (D) Kaplan-Meier estimates of survival time in 2 ESCC patient cohorts and combined samples by different TNFR1 protein levels in tumors with HR = 2.548 (95% CI = 1.419–6.323). (E) A proposed model for the regulatory mechanism of the METTL3-m^{6}A-TNFR1-ATXN2-NF-κB/MAPK signaling axis in the tumorigenesis and progression of ESCC. p values were calculated by the two-sided Mann-Whitney test in (A) and (C). Two-sided log rank test was used in (B) and (D) (*$p < 0.05$, **$p < 0.01$, and ***$p < 0.001$).

a promising potential way to increase clinical benefit that needs further exploration in the future.

We acknowledge some limitations in the present study. Here, we reported that a potential m^{6}A mediator ATXN2 regulates TNFR1 translation during ESCC development. However, integrated analysis of published ATXN2 PAR-CLIP-seq (HEK293T cells) data and m^{6}A-seq data of our ESCC tissues revealed a high binding intensity for ATXN2 centered at m^{6}A residues and vice versa for m^{6}A in the global transcriptome level (Figure 3F), but whether and how ATXN2 regulates other m^{6}A-modified transcripts in ESCC or other types of cancer should be investigated in the future. In addition, some biological processes such as apoptosis, Hippo, and mammalian target of rapamycin (mTOR) pathways were also regulated by abnormal methylation (Figure 1E). We cannot deny that the important roles of these oncogenic pathways are affected by elevated m^{6}A modification in ESCC development. However, we observed that many transcripts were hypermethylated and that these transcripts were extensively involved in the TNFR1-mediated MAPK and NF-κB regulatory network. Therefore, we think that the systematic regulatory network may play more important role in ESCC than other biological processes such as apoptosis, Hippo, and mTOR-mediated pathways. The detail mechanism of methylation dysregulation in other biological processes in ESCC need to be further explored. In summary, evidence is emerging that the aberrant m^{6}A modification of TNFR1 plays important roles in the initiation and progression of ESCC through ATXN2-induced posttranscriptional regulation, which is an important oncogenic mechanism for ESCC.

MATERIALS AND METHODS

Study subjects

Surgically removed ESCC tumors and the corresponding adjacent normal tissue samples (N = 281) were obtained from the Sun Yat-sen University Cancer Center. Of 281 paired samples, 8 were used for m^{6}A-seq (collected from 2015 to 2017; Table S5); 215 (Table S2) and the remaining 58 paired samples (Table S3) were used for qRT-PCR and IHC analysis, respectively (collected from 2012 to 2014). The diagnosis of ESCC was confirmed by pathological examination, and tumor stage was defined according to the 7th edition of the American Joint Committee on Cancer (AJCC) Cancer Staging System. All ESCC tissues and paired adjacent normal tissues were obtained from ESCC patients during surgery and were stored in liquid nitrogen. Clinical information about the ESCC patients was obtained from their medical records. The survival time of the patients was recorded from the date of diagnosis to the date of last follow-up or death. Follow-up information was obtained from telephone calls, medical records, or outpatient visits. The study was approved by the institutional review board of the SYSUCC and informed consent was obtained from each participant.

Tissue RNA isolation

Total RNA was isolated from ESCC tissues and adjacent normal tissues with TRIzol reagent (Invitrogen). The RNA samples were quantified by measuring absorbance at 260 nm with a UV spectrophotometer, and only analytes with an RNA integrity number (RIN) ≥ 7.0 were used in further experiments.

m6A-seq

Total RNA was digested with DNase I, and rRNA content was reduced by using RiboMinus (Illumina). RNA fragmentation and m^{6}A-IP were performed according to previously published protocols.[41] Sequencing for the m^{6}A-IP was performed using an Illumina HiSeq2500 SE50, and sequencing for the input was performed on an Illumina HiSeq2500 machine in pair-read mode with 150 bp per read.

m6A-seq data analyses

The input reads were trimmed to the same length as the m^{6}A-IP reads using fastx_trimmer from FASTX-Toolkit (http://hannonlab.cshl.edu/fastx_toolkit/). The 50-bp m^{6}A-IP reads and input reads were mapped to the hg19 genome using STAR.[42] We used MACS2[43] and MeTPeak[44] to identify peaks for 8 pairs of samples, and the cutoff of the p value for MACS2[43] was 1e−6. For each sample, only m^{6}A peaks identified by both peak-calling software programs were retained and merged using BEDTools.[45] To avoid false positives, the peaks detected in at least 2 samples were retained, and the 5′ UTR peaks with transcription start site (TSS) “A” and “BCA” motifs were filtered out. The m^{6}A annotation was performed with the human annotation file (GENCODE, version 27) downloaded from the GENCODE database: https://www.gencodegenes.org/.[46] We compared ESCC m^{6}A peaks to the recorded m^{6}A sites in RMBase[22] using IntersectBed. Homer was used to search the motif enriched in m^{6}A peaks. The relative m^{6}A level for each m^{6}A peak was quantified as previously described.[47] The read coverage of each peak in m^{6}A-IP and input were calculated using Multicov from BEDTools[45] and normalized by the RPKM (reads per kilobase million) method. The enrichment of each m^{6}A peak was the ratio of IP RPKM to input RPKM. To assess global changes to m^{6}A methylation, the enrichment values of m^{6}A peaks were averaged over all of the tumors or paired adjacent normal samples. Furthermore, we calculated the enrichment changes of the m^{6}A peak in each ESCC tissue versus the corresponding normal tissue. Annotations for pathways were performed according to the Kyoto Encyclopedia of Genes and Genomes (KEGG) database: https://www.genome.jp/kegg/[48]

Cell lines and cell culture

Human ESCC cell lines KYSE30 and EC109 were obtained from Dr. Xinyuan Guan at the Sun Yat-sen University Cancer Center. Human embryonic kidney cell line 293T was purchased from the Cell Bank of Type Culture Collection of the Chinese Academy of Sciences Shanghai Institute of Biochemistry and Cell Biology. Cells were maintained in RPMI 1640 (KYSE30 and EC109) or DMEM (293T) medium supplemented with 10% fetal bovine serum (FBS) at 37°C and 5% CO_2. Cells were authenticated by DNA fingerprinting analysis using short-tandem repeat (STR) markers and were not infected with mycoplasma.

m6A RNA immunoprecipitation followed by quantitative real-time-PCR

Fragmented RNA from the ESCC tissue and cell lines was immunoprecipitated by anti-m^{6}A antibody, and then the purified m^{6}A-containing RNA was reverse transcribed and amplified; m^{6}A methylation changes in target genes were quantified as described previously.[49] The primer sequences are shown in Table S6.

Single-base elongation and ligation-based quantitative PCR amplification

The SELECT quantitative PCR method was performed as previous reported.[27] Briefly, 2 μg total RNA was mixed with 40 nM primers (forward and reverse) and 5 μM deoxynucleoside triphosphates (dNTPs) in 17 μL 1 × CutSmart buffer (NEB). The RNA and primers were annealed by incubating at a temperature gradient: 90°C for 1 min, 80°C for 1 min, 70°C for 1 min, 60°C for 1 min, 50°C for 1 min, and 40°C for 6 min. The annealing product was subsequently mixed with 0.5 U SplintR ligase, 10 nM ATP, and 3 μL 0.01 U Bst 2.0 DNA polymerase, incubated at 40°C for 20 min, denatured at 80°C for 20 min, and kept at 4°C. A total of 2 μL of the final reaction mixture was used to test the ligation efficiency by quantitative PCR with the SELECT primers listed in Table S6.

Small interfering RNAs (siRNAs) and guide RNAs

siRNA targeting *ATXN2* was purchased from GenePharma (Table S7). For the dm^{6}ACRISPR system, two gRNAs were designed according to the m^{6}A site of TNFR1 (Table S6) and were subjected to NCBI BLAST: https://blast.ncbi.nlm.nih.gov/Blast.cgi to prevent alignment with nontarget RNAs in the human genome.

Plasmids, lentivirus production, and transduction

To construct lentiviral vector expressing human *METTL3* (NM_019852.5) and *TNFRSF1A* (NM_001065.4), the full lengths of *METTL3* and *TNFRSF1A* protein coding sequences were commercially synthesized and subcloned into pLenti-CMV-Puro vector (Obio Technology) and pLVX-EF1a-Puro-CMV-MCS (Umine Biotechnology). Short hairpin RNA (shRNA) specifically targeting METTL3 and TNFR1 (Table S7) was synthesized and subcloned into pLKD-U6-MCS-CMV-Puro (Umine Biotechnology) lentiviral shRNA vectors. WT or catalytic mutant (aa395–398, DPPW→APPA) METTL3 was subcloned into pLenti-CMV-MCS-PGK-Puro lentiviral expression vector (Obio Technology).

The PspCas13b-ALKBH5 plasmid was inserted into the pLVX-Tet 3G lentiviral expression vector (Umine Biotechnology) and its expression was measured after doxycycline induction. gRNAs were subcloned into pLKD-U6-Cas13bgRNA-CMV-Blasticidin to synthesize gRNA-containing plasmids. 293T cells produced lentiviruses after transfection with the vector plasmid and the lentiviral vector packaging system (Obio Technology), and ESCC cells were infected with concentrated lentiviral particles in the presence of polybrene. The expression of target genes in infected cells was detected by quantitative real-time-PCR.

Quantitative real-time-PCR

Total RNA was extracted from ESCC tissue and cell lines using TRIzol reagent. cDNA was synthesized using the RevertAid First Strand cDNA Synthesis Kit (Thermo Fisher Scientific) and quantified on a

Roche Light Cycler 480 II using the SYBR-Green method.[50] β-Actin was used as the internal control. The relative expression of RNAs was calculated by normalizing to the control. The primer sequences are shown in Table S6.

Protein stability assay

Protein stability of targets in METTL3 or ATXN2 knockdown ESCC cells and siControl cells was achieved via the incubation of cycloheximide (CHX, final concentration 10 μg/mL) during the indicated times. The protein level of TNFR1 was determined by western blot analysis.

Western blot assays

Protein from ESCC tissues or cells was extracted using detergent-containing lysis buffer. Total protein (30 μg) was subjected to SDS-PAGE and transferred to polyvinylidene difluoride (PVDF) membranes (Millipore, Billerica). The membranes were incubated overnight at 4°C with a specific antibody and visualized with a Phototope Horseradish Peroxidase Western Blot Detection kit (Thermo Fisher Scientific). Detailed information about the antibody against target proteins is shown in Table S8.

Immunohistochemical staining

Paraffin-embedded tissues from ESCC patients were used for IHC analysis. Detailed information about the antibodies against METTL3, TNFR1, and Ki67 are shown in Table S8. The staining intensity was estimated as negative (0), weak (1), moderate (2), and strong (3). The extent of staining, defined as the percentage of positively stained cells, was graded as 1 (≤25%), 2 (26%–50%), 3 (51%–75%), or 4 (>75%). The total immunoreactive score (IRS) was calculated by multiplying the score of intensity and extent.

ATXN2 CLIP quantitative real-time-PCR

ESCC cells were washed with ice-cold PBS and irradiated with 365 nm UV light to induce crosslinking. Nuclear extracts were sonicated by DNase I and low-dilution RNase I treatments. Dynabeads protein A/G (Millipore) conjugated with anti-ATXN2 antibody was incubated with extraction and rotated overnight at 4°C. Then, after treatment with proteinase K, extraction with acidic phenol/chloroform and precipitation with ethanol, the bound RNAs were detected by quantitative real-time-PCR. The primers are listed in Table S6.

RNA pulldown and mass spectrometry analysis

RNA pulldown was performed with the Pierce Magnetic RNA-Protein Pull-Down Kit (20164, Thermo Fisher Scientific); biotin-labeled RNA probes with or without m^6A modification were synthesized and incubated with cellular protein extracts from KYSE30 cells. After adding streptavidin beads, recovered total proteins were subjected to mass spectrometry analysis. To identify the potential m^6A mediators, we used the following strategies: unique peptides >12, ratio of average label-free quantitation (LFQ) intensity of TNFR1[m^6A] and TNFR1 [A] > 1.5. The proteins identified by mass spectrometry are listed in Table S4.

RNA electrophoretic mobility shift assays

Assays were performed using the LightShift Chemiluminescent RNA EMSA Kit (Thermo Fisher Scientific), and the biotin-labeled RNA probes were synthesized by Ruibiotech (Beijing, China). Briefly, 1-μL RNA probes (4 nM final concentration) were incubated in binding buffer (10 mM HEPES pH 7.3, 20 mM KCl, 1 mM $MgCl_2$, 1 mM dithiothreitol (DTT), 5% glycerol, and 40 U/mL RNasin) with different concentrations of recombinant ATXN2 proteins at room temperature for 20 min. The RNA-protein mixtures were separated in 8% native polyacrylamide gels at 4°C for 60 min. Proteins were transferred from the gels to a nylon membrane, cross-linked to the membrane using the UVP Crosslinker (120 mJ/cm^2 of 254 nm UV), and detected by chemiluminescence.

Polysome profiling

ESCC cells were treated with 100 μg/mL CHX for 7 min, lysed on ice in lysis buffer (5 mM Tris-HCl, 2.5 mM $MgCl_2$, 1.5 mM KCl, protease inhibitor cocktail, 5 μL 10 mg/mL CHX, 1 μL 1 M DTT, 100 U RNase inhibitor), and vortexed for 15 s. After adding 25 μL 10% Triton X-100 and 25 μL 10% sodium deoxycholate, ESCC cells were vortexed for 10 s again. Cell lysates were incubated on ice for 10 min and centrifuged at 4°C for 7 min at 16,000 rpm. Ten percent of the lysate was used to determine cytosolic steady-state RNA levels. The supernatant was loaded on top of a 5%–50% sucrose gradient and centrifuged at 4°C for 2 h at 222,228 rpm (Beckman). The gradients were collected by monitoring RNA absorbance at 254 nm with an ISCO fractionator, and total RNA was purified and analyzed by quantitative real-time-PCR.

Cell viability and colony-formation assays

For cell viability assays, cells were seeded in 96-well plates (2,000 cells per well), and cell viability was measured using the CCK-8 kit (Dojindo Laboratories). For the colony-formation assay, cells were seeded in 12-well plates (500 cells per well). After 2 weeks, colonies were stained using Crystal Violet and counted.

Cell invasion and migration assays

Invasion assays were performed in a 24-well culture plate, and 8-μm pore inserts were coated with 30 μg Matrigel (BD Biosciences). Cells (1×10^5) were added to the coated filters in 200-μL serum-free medium, and 1640 medium supplemented with 20% FBS was added to the lower chamber. After incubation for 20 h at 37°C in 5% CO_2, cells that migrated through the filters were fixed with methanol, stained with Crystal Violet, and photographed. Cell numbers in three random fields were counted. The migration assays were performed according to a similar protocol without coating with Matrigel.

Animal experiments

Animal experiments were approved by the Institutional Animal Care and Use Committee of the Sun Yat-sen University Cancer Center. Female BALB/c nude mice (4–5 weeks old) were housed with a 12/12 h light/dark cycle and 50%–70% humidity, and food and water were provided *ad libitum*. For the subcutaneous xenograft tumor model, ESCC cells transduced with lentivirus expressing TNFR1 Control,

TNFR1-Overexpression, shControl, shTNFR1-1, or shTNFR1-2 were injected subcutaneously into the dorsal flanks of BALB/c nude mice (2×10^6 cells in 0.1 mL PBS/mouse) (N = 5 in each group). Tumor size was measured once every week and calculated according to the following formula: volume = length $\times$ width^2 $\times$ 0.5.

Public data processing

To examine the expression of METTL3 in human ESCC, a public dataset of ESCC (N = 179, GSE53625)[25] was downloaded from the GEO. A two-sided Student's t test was used for comparing the METTL3 expression levels between ESCC and paired normal tissue (N = 179). We obtained the protein ATXN2 binding sites from the POSTAR2 database: http://lulab.life.tsinghua.edu.cn/POSTAR/[51] and the exact m^{6}A sites of TNFR1 from 2 independent public datasets (GSM4084010[52]; GSM1828594,[53] miCLIP-seq).

Statistical analysis

The results are presented as the mean ± standard error of the mean (SEM) of at least 3 biological replicates. A two-sided Student's t test was performed to compare two means between groups, and data with abnormal distribution were assessed using a nonparametric test. The correlation between two continuous variables was calculated using Pearson's correlations, and $p < 0.05$ and $|r| > 0.30$ were considered significant. For the survival analysis, Kaplan-Meier analysis using the R package Survival was used to estimate the distribution of the survival time. The log rank test was performed to compare differences between survival distributions. Statistical analyses were performed using GraphPad Prism 8.0 software (GraphPad) or the R programming environment (version 3.6.0; R Foundation for Statistical Computing).

Data availability

The accession number for the m^{6}A-seq data reported in this paper is HRA000590 (https://bigd.big.ac.cn/gsa-human/browse/HRA000590).

SUPPLEMENTAL INFORMATION

Supplemental information can be found online at https://doi.org/10.1016/j.ymthe.2022.01.006.

ACKNOWLEDGMENTS

This study was supported by National key R&D Program of China (2021YFA1302100, to J. Zheng), the Program for Guangdong Introducing Innovative and Entrepreneurial Teams (2017ZT07S096, to D.L.), Natural Science Foundation of China (U1601229, to D.L.), National Young Top-notch Talent Support Program (to J. Zheng), Sun Yat-sen University Intramural Funds (to D.L. and J. Zheng).

AUTHOR CONTRIBUTIONS

X.H., J. Zheng and D.L. conceptualized and supervised the research. X.H., L. Zeng, H.Z., J.D., and L.P. performed most of the experiments. G.W., J. Zhang, J.S., and Y.Z. performed the animal experiments. R.L., Y.Y., S.D., and Z.Z. were engaged in the biostatistics and bioinformatics analysis. S.Z., R.B., L. Zhuang, and M.L. were responsible for patient recruitment, biospecimen, and clinical data collection. D.L., J. Zheng, X.H., and R.L. drafted the manuscript.

DECLARATION OF INTERESTS

The authors declare no competing interests.

REFERENCES

1. Chen, W., Zheng, R., Baade, P.D., Zhang, S., Zeng, H., Bray, F., Jemal, A., Yu, X.Q., and He, J. (2016). Cancer statistics in China, 2015. CA Cancer J. Clin. *66*, 115–132.
2. Lin, Y., Totsuka, Y., Shan, B., Wang, C., Wei, W., Qiao, Y., Kikuchi, S., Inoue, M., Tanaka, H., and He, Y. (2017). Esophageal cancer in high-risk areas of China: research progress and challenges. Ann. Epidemiol. *27*, 215–221.
3. Lin, D.C., Wang, M.R., and Koeffler, H.P. (2018). Genomic and epigenomic aberrations in esophageal squamous cell carcinoma and implications for patients. Gastroenterology *154*, 374–389.
4. Adams, J.M., and Cory, S. (1975). Modified nucleosides and bizarre 5'-termini in mouse myeloma mRNA. Nature *255*, 28–33.
5. Zaccara, S., Ries, R.J., and Jaffrey, S.R. (2019). Reading, writing and erasing mRNA methylation. Nat. Rev. Mol. Cell Biol. *20*, 608–624.
6. Yue, Y., Liu, J., Cui, X., Cao, J., Luo, G., Zhang, Z., Cheng, T., Gao, M., Shu, X., Ma, H., et al. (2018). VIRMA mediates preferential m^{6}A mRNA methylation in 3'UTR and near stop codon and associates with alternative polyadenylation. Cell Discov *4*, 10.
7. Xiao, W., Adhikari, S., Dahal, U., Chen, Y.S., Hao, Y.J., Sun, B.F., Sun, H.Y., Li, A., Ping, X.L., Lai, W.Y., et al. (2016). Nuclear m^{6}A reader YTHDC1 regulates mRNA splicing. Mol. Cell *61*, 507–519.
8. Liu, N., Dai, Q., Zheng, G., He, C., Parisien, M., and Pan, T. (2015). N^6-methyladenosine-dependent RNA structural switches regulate RNA-protein interactions. Nature *518*, 560–564.
9. Alarcón, C.R., Goodarzi, H., Lee, H., Liu, X., Tavazoie, S., and Tavazoie, S.F. (2015). HNRNPA2B1 is a mediator of m^{6}A-dependent nuclear RNA processing events. Cell *162*, 1299–1308.
10. Shi, H., Wang, X., Lu, Z., Zhao, B.S., Ma, H., Hsu, P.J., Liu, C., and He, C. (2017). YTHDF3 facilitates translation and decay of N^6-methyladenosine-modified RNA. Cell Res. *27*, 315–328.
11. Wang, X., Lu, Z., Gomez, A., Hon, G.C., Yue, Y., Han, D., Fu, Y., Parisien, M., Dai, Q., Jia, G., et al. (2014). N^6-methyladenosine-dependent regulation of messenger RNA stability. Nature *505*, 117–120.
12. Ma, J.Z., Yang, F., Zhou, C.C., Liu, F., Yuan, J.H., Wang, F., Wang, T.T., Xu, Q.G., Zhou, W.P., and Sun, S.H. (2017). METTL14 suppresses the metastatic potential of hepatocellular carcinoma by modulating N^6-methyladenosine-dependent primary microRNA processing. Hepatology *65*, 529–543.
13. Weng, H., Huang, H., Wu, H., Qin, X., Zhao, B.S., Dong, L., Shi, H., Skibbe, J., Shen, C., Hu, C., et al. (2018). METTL14 inhibits hematopoietic stem/progenitor differentiation and promotes leukemogenesis via mRNA m^{6}A modification. Cell Stem Cell *22*, 191–205.
14. Liu, Z.G. (2005). Molecular mechanism of TNF signaling and beyond. Cell Res *15*, 24–27.
15. Smith, C.A., Farrah, T., and Goodwin, R.G. (1994). The TNF receptor superfamily of cellular and viral proteins: activation, costimulation, and death. Cell *76*, 959–962.
16. Gregory, A.P., Dendrou, C.A., Attfield, K.E., Haghikia, A., Xifara, D.K., Butter, F., Poschmann, G., Kaur, G., Lambert, L., Leach, O.A., et al. (2012). TNF receptor 1 genetic risk mirrors outcome of anti-TNF therapy in multiple sclerosis. Nature *488*, 508–511.
17. Knight, B., Yeoh, G.C., Husk, K.L., Ly, T., Abraham, L.J., Yu, C., Rhim, J.A., and Fausto, N. (2000). Impaired preneoplastic changes and liver tumor formation in tumor necrosis factor receptor type 1 knockout mice. J. Exp. Med. *192*, 1809–1818.
18. Arnott, C.H., Scott, K.A., Moore, R.J., Robinson, S.C., Thompson, R.G., and Balkwill, F.R. (2004). Expression of both TNF-α receptor subtypes is essential for optimal skin tumour development. Oncogene *23*, 1902–1910.

19. Oshima, H., Ishikawa, T., Yoshida, G.J., Naoi, K., Maeda, Y., Naka, K., Ju, X., Yamada, Y., Minamoto, T., Mukaida, N., et al. (2014). TNF-α/TNFR1 signaling promotes gastric tumorigenesis through induction of Noxo1 and Gna14 in tumor cells. Oncogene *33*, 3820–3829.

20. Popivanova, B.K., Kitamura, K., Wu, Y., Kondo, T., Kagaya, T., Kaneko, S., Oshima, M., Fujii, C., and Mukaida, N. (2008). Blocking TNF-α in mice reduces colorectal carcinogenesis associated with chronic colitis. J. Clin. Invest. *118*, 560–570.

21. Dominissini, D., Moshitch-Moshkovitz, S., Schwartz, S., Salmon-Divon, M., Ungar, L., Osenberg, S., Cesarkas, K., Jacob-Hirsch, J., Amariglio, N., Kupiec, M., et al. (2012). Topology of the human and mouse m^6A RNA methylomes revealed by m^6A-seq. Nature *485*, 201–206.

22. Xuan, J.J., Sun, W.J., Lin, P.H., Zhou, K.R., Liu, S., Zheng, L.L., Qu, L.H., and Yang, J.H. (2018). RMBase v2.0: deciphering the map of RNA modifications from epitranscriptome sequencing data. Nucleic Acids Res. *46*, 327–334.

23. Hehlgans, T., and Pfeffer, K. (2005). The intriguing biology of the tumour necrosis factor/tumour necrosis factor receptor superfamily: players, rules and the games. Immunology *115*, 1–20.

24. Baud, V., and Karin, M. (2001). Signal transduction by tumor necrosis factor and its relatives. Trends Cell Biol *11*, 372–377.

25. Li, J., Chen, Z., Tian, L., Zhou, C., He, M.Y., Gao, Y., Wang, S., Zhou, F., Shi, S., Feng, X., et al. (2014). LncRNA profile study reveals a three-lncRNA signature associated with the survival of patients with oesophageal squamous cell carcinoma. Gut *63*, 1700–1710.

26. Lin, S., Choe, J., Du, P., Triboulet, R., and Gregory, R.I. (2016). The m^6A methyltransferase METTL3 promotes translation in human cancer cells. Mol. Cell *62*, 335–345.

27. Xiao, Y., Wang, Y., Tang, Q., Wei, L., Zhang, X., and Jia, G. (2018). An elongation- and ligation-based qPCR amplification method for the radiolabeling-free detection of locus-specific N^6-methyladenosine modification. Angew. Chem. Int. Ed. Engl. *57*, 15995–16000.

28. Li, J., Chen, Z., Chen, F., Xie, G., Ling, Y., Peng, Y., Lin, Y., Luo, N., Chiang, C.M., and Wang, H. (2020). Targeted mRNA demethylation using an engineered dCas13b-ALKBH5 fusion protein. Nucleic Acids Res. *48*, 5684–5694.

29. Ting, A.T., and Bertrand, M.J.M. (2016). More to life than NF-κB in TNFR1 signaling. Trends Immunol. *37*, 535–545.

30. Egusquiaguirre, S.P., Yeh, J.E., Walker, S.R., Liu, S., and Frank, D.A. (2018). The STAT3 target gene TNFRSF1A modulates the NF-κB pathway in breast cancer cells. Neoplasia *20*, 489–498.

31. Komori, J., Marusawa, H., Machimoto, T., Endo, Y., Kinoshita, K., Kou, T., Haga, H., Ikai, I., Uemoto, S., and Chiba, T. (2008). Activation-induced cytidine deaminase links bile duct inflammation to human cholangiocarcinoma. Hepatology *47*, 888–896.

32. Locksley, R.M., Killeen, N., and Lenardo, M.J. (2001). The TNF and TNF receptor superfamilies: integrating mammalian biology. Cell *104*, 487–501.

33. Liu, Z.G., Hsu, H., Goeddel, D.V., and Karin, M. (1996). Dissection of TNF receptor 1 effector functions: JNK activation is not linked to apoptosis while NF-κB activation prevents cell death. Cell *87*, 565–576.

34. Wang, X., Zhao, B.S., Roundtree, I.A., Lu, Z., Han, D., Ma, H., Weng, X., Chen, K., Shi, H., and He, C. (2015). N^6-methyladenosine modulates messenger RNA translation efficiency. Cell *161*, 1388–1399.

35. Li, A., Chen, Y.S., Ping, X.L., Yang, X., Xiao, W., Yang, Y., Sun, H.Y., Zhu, Q., Baidya, P., Wang, X., et al. (2017). Cytoplasmic m^6A reader YTHDF3 promotes mRNA translation. Cell Res. *27*, 444–447.

36. Pulst, S.M., Nechiporuk, A., Nechiporuk, T., Gispert, S., Chen, X.N., Lopes-Cendes, I., Pearlman, S., Starkman, S., Orozco-Diaz, G., Lunkes, A., et al. (1996). Moderate expansion of a normally biallelic trinucleotide repeat in spinocerebellar ataxia type 2. Nat. Genet. *14*, 269–276.

37. Ralser, M., Albrecht, M., Nonhoff, U., Lengauer, T., Lehrach, H., and Krobitsch, S. (2005). An integrative approach to gain insights into the cellular function of human ataxin-2. J. Mol. Biol. *346*, 203–214.

38. Kozlov, G., Safaee, N., Rosenauer, A., and Gehring, K. (2010). Structural basis of binding of P-body-associated proteins GW182 and ataxin-2 by the Mlle domain of poly(A)-binding protein. J. Biol. Chem. *285*, 13599–13606.

39. Elden, A.C., Kim, H.J., Hart, M.P., Chen-Plotkin, A.S., Johnson, B.S., Fang, X., Armakola, M., Geser, F., Greene, R., Lu, M.M., et al. (2010). Ataxin-2 intermediate-length polyglutamine expansions are associated with increased risk for ALS. Nature *466*, 1069–1075.

40. Huang, H., Weng, H., and Chen, J. (2020). m^6A modification in coding and non-coding RNAs: roles and therapeutic implications in cancer. Cancer Cell *37*, 270–288.

41. Zhang, C., Chen, Y., Sun, B., Wang, L., Yang, Y., Ma, D., Lv, J., Heng, J., Ding, Y., Xue, Y., et al. (2017). m^6A modulates haematopoietic stem and progenitor cell specification. Nature *549*, 273–276.

42. Dobin, A., Davis, C.A., Schlesinger, F., Drenkow, J., Zaleski, C., Jha, S., Batut, P., Chaisson, M., and Gingeras, T.R. (2013). STAR: ultrafast universal RNA-seq aligner. Bioinformatics *29*, 15–21.

43. Zhang, Y., Liu, T., Meyer, C.A., Eeckhoute, J., Johnson, D.S., Bernstein, B.E., Nusbaum, C., Myers, R.M., Brown, M., Li, W., and Liu, X.S. (2008). Model-based analysis of ChIP-seq (MACS). Genome Biol. *9*, 137.

44. Cui, X., Meng, J., Zhang, S., Chen, Y., and Huang, Y. (2016). A novel algorithm for calling mRNA m^6A peaks by modeling biological variances in MeRIP-Seq data. Bioinformatics *32*, 378–385.

45. Quinlan, A.R., and Hall, I.M. (2010). BEDTools: a flexible suite of utilities for comparing genomic features. Bioinformatics *26*, 841–842.

46. Frankish, A., Diekhans, M., Ferreira, A.M., Johnson, R., Jungreis, I., Loveland, J., Mudge, J.M., Sisu, C., Wright, J., Armstrong, J., et al. (2019). GENCODE reference annotation for the human and mouse genomes. Nucleic Acids Res. *47*, 766–773.

47. Schwartz, S., Mumbach, M.R., Jovanovic, M., Wang, T., Maciag, K., Bushkin, G.G., Mertins, P., Ter-Ovanesyan, D., Habib, N., Cacchiarelli, D., et al. (2014). Perturbation of m^6A writers reveals two distinct classes of mRNA methylation at internal and 5' sites. Cell Rep. *8*, 284–296.

48. Kanehisa, M., Furumichi, M., Tanabe, M., Sato, Y., and Morishima, K. (2017). KEGG: new perspectives on genomes, pathways, diseases and drugs. Nucleic Acids Res. *45*, 353–361.

49. Zhang, J., Bai, R., Li, M., Ye, H., Wu, C., Wang, C., Li, S., Tan, L., Mai, D., Li, G., et al. (2019). Excessive miR-25-3p maturation via N^6-methyladenosine stimulated by cigarette smoke promotes pancreatic cancer progression. Nat. Commun. *10*, 1858.

50. Schneeberger, C., Speiser, P., Kury, F., and Zeillinger, R. (1995). Quantitative detection of reverse transcriptase-PCR products by means of a novel and sensitive DNA stain. PCR Methods Appl. *4*, 234–238.

51. Zhu, Y., Xu, G., Yang, Y.T., Xu, Z., Chen, X., Shi, B., Xie, D., Lu, Z.J., and Wang, P. (2019). POSTAR2: deciphering the post-transcriptional regulatory logics. Nucleic Acids Res. *47*, 203–211.

52. Chai, P., Yu, J., Jia, R., Wen, X., Ding, T., Zhang, X., Ni, H., Jia, R., Ge, S., Zhang, H., and Fan, X. (2020). Generation of onco-enhancer enhances chromosomal remodeling and accelerates tumorigenesis. Nucleic Acids Res. *48*, 12135–12150.

53. Ke, S., Alemu, E.A., Mertens, C., Gantman, E.C., Fak, J.J., Mele, A., Haripal, B., Zucker-Scharff, I., Moore, M.J., Park, C.Y., et al. (2015). A majority of m^6A residues are in the last exons, allowing the potential for 3' UTR regulation. Genes Dev. *29*, 2037–2053.

YMTHE, Volume *30*

Supplemental Information

ATXN2-mediated translation of TNFR1 promotes esophageal squamous cell carcinoma via m^{6}A-dependent manner

Rui Li, Lingxing Zeng, Hongzhe Zhao, Junge Deng, Ling Pan, Shaoping Zhang, Guandi Wu, Ying Ye, Jialiang Zhang, Jiachun Su, Yanfen Zheng, Shuang Deng, Ruihong Bai, Lisha Zhuang, Mei Li, Zhixiang Zuo, Dongxin Lin, Jian Zheng, and Xudong Huang

Li et al_Supplementary Figure 1

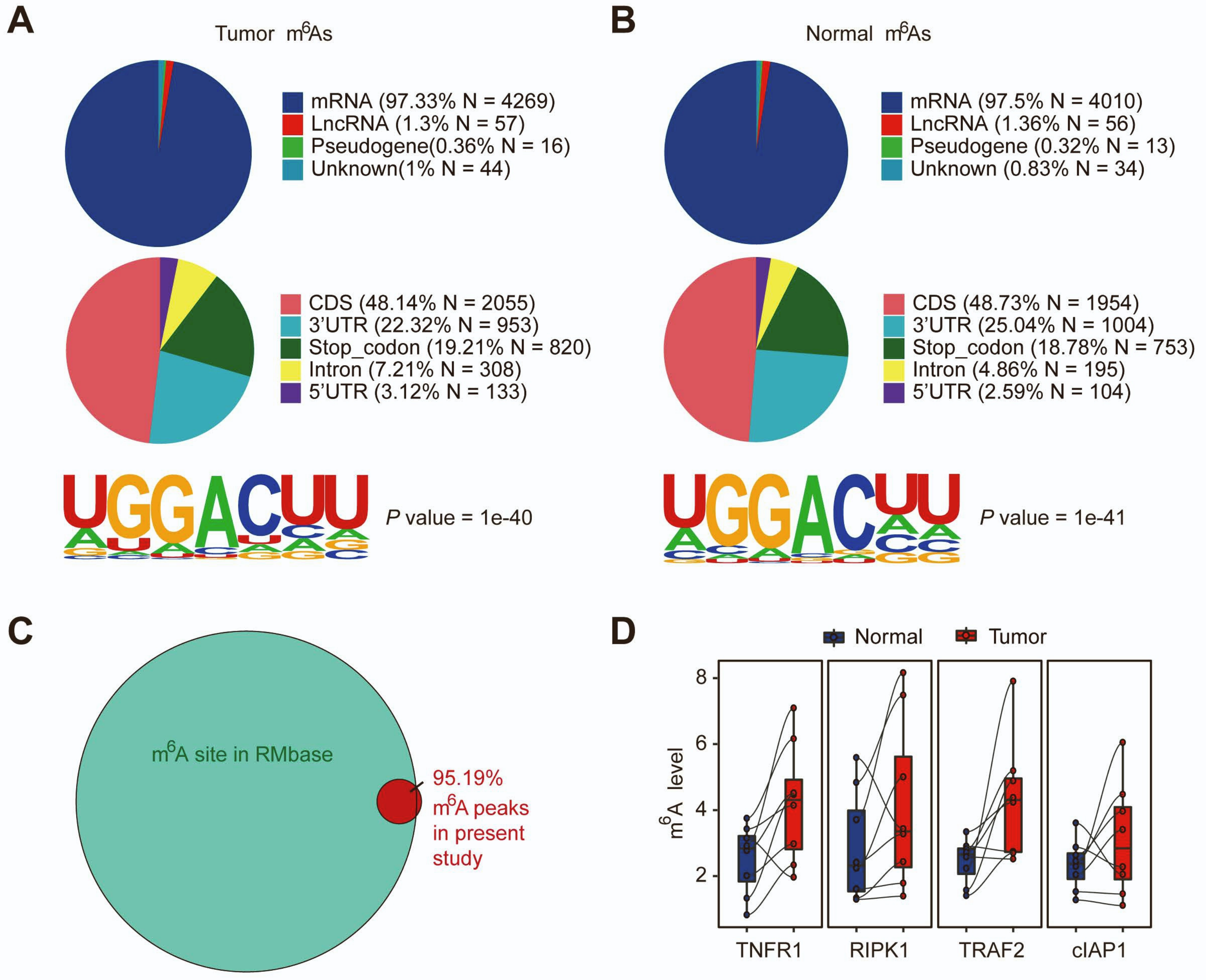

Figure S1. Distribution of RNA m^6A modification in human ESCC. Related to Figure 1.

(A, B) The distribution of annotated m^6A-modified transcripts in different kinds of RNA (*upper panel*: mRNA, lncRNA, pseudogene and others) and a variety of regions (*middle panel*: 5'UTR, CDS, 3'UTR, stop codon and intron). Sequence logo showing the top rank motif detected by Homer (*lower panel*). (C) The Venn plot shows overlap between the m^6A peaks in our m^6A-seq analysis and the m^6A sites in the RMBase database. (D) The m^6A modification level of key molecules (TNFR1, RIPK1, TRAF2 and cIAP1) of TNFR1 signaling in 8 ESCC tumors and paired adjacent normal samples according to m^6A-seq.

Li et al_Supplementary Figure 2

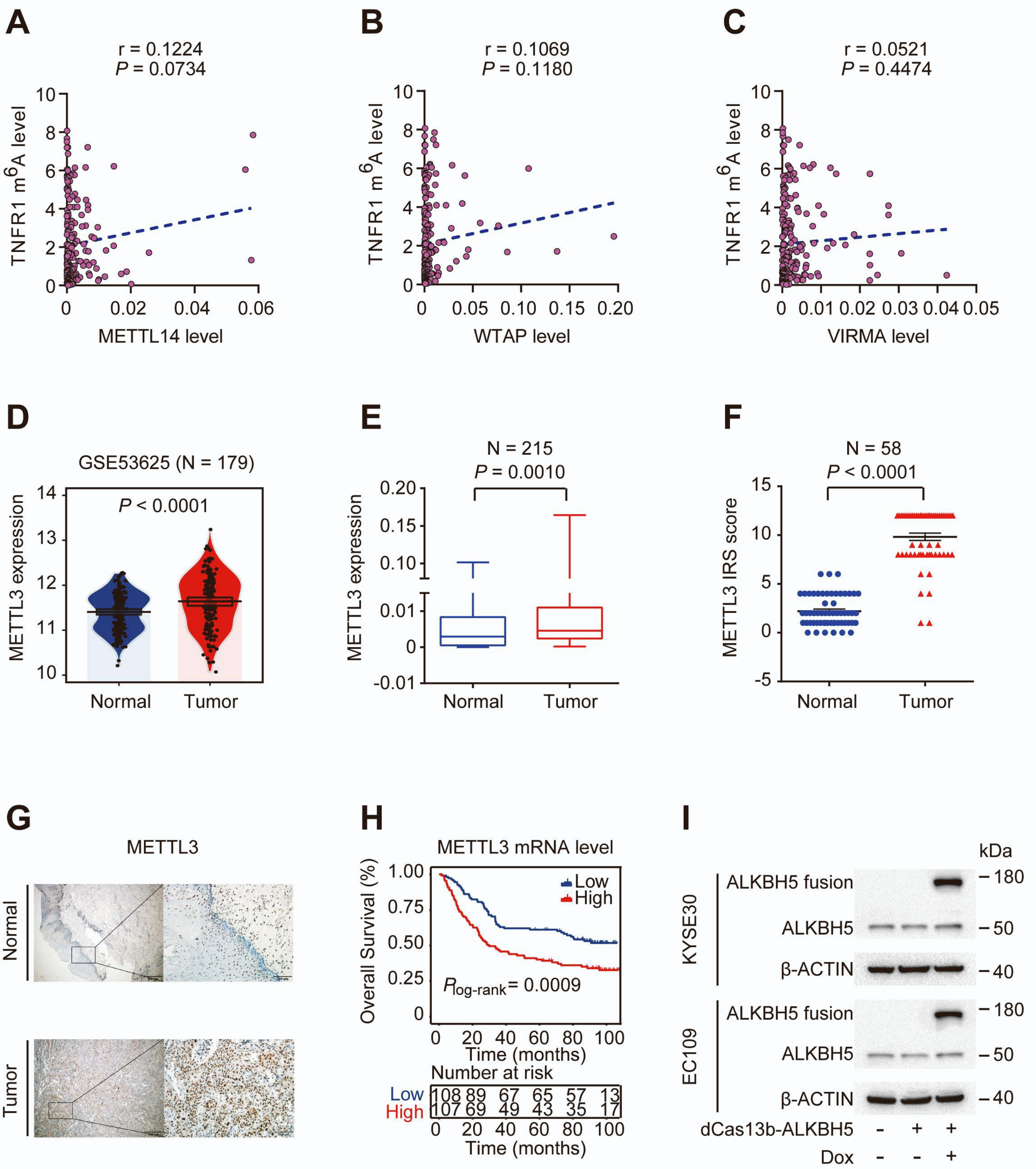

Figure S2. METTL3 expression is significantly upregulated in ESCC. Related to Figure 2.

(A-C) Spearman's correlation analysis between TNFR1 m^6A levels and the mRNA levels of common m^6A writers (METTL14, A; WTAP, B; VIRMA, C) in ESCC tumor tissues (N = 215). (D) METTL3 mRNA expression in ESCC tumors and paired adjacent normal tissues from a public dataset (GSE53625, N = 179). (E) Expression levels of METTL3 RNA in ESCC samples compared with paired normal tissues (SYSUCC cohort, N = 215). (F, G) Quantification of IHC staining (F, SYSUCC cohort, N = 58) and representative IHC images of METTL3 protein levels in ESCC tumors and paired adjacent normal tissues (G). Scale bar, 500 μm (left) and 100 μm (right). (H) Kaplan-Meier estimates of survival time of ESCC patients in SYSUCC Cohort (N = 215) by different METTL3 levels, with the adjusted HRs (95% CI) for death of high METTL3 level being 1.840 (1.284–2.635). (I) Western blot showing the expression of the dCas13b-ALKBH5 fusion protein in ESCC cells with or without doxycycline induction. *P* values were calculated by two-sided paired Wilcoxon signed-rank test in (D), (E) and (F), and two-sided log-rank test was used in (H) (*$P < 0.05$, **$P < 0.01$ and ***$P < 0.001$).

Li et al_Supplementary Figure 3

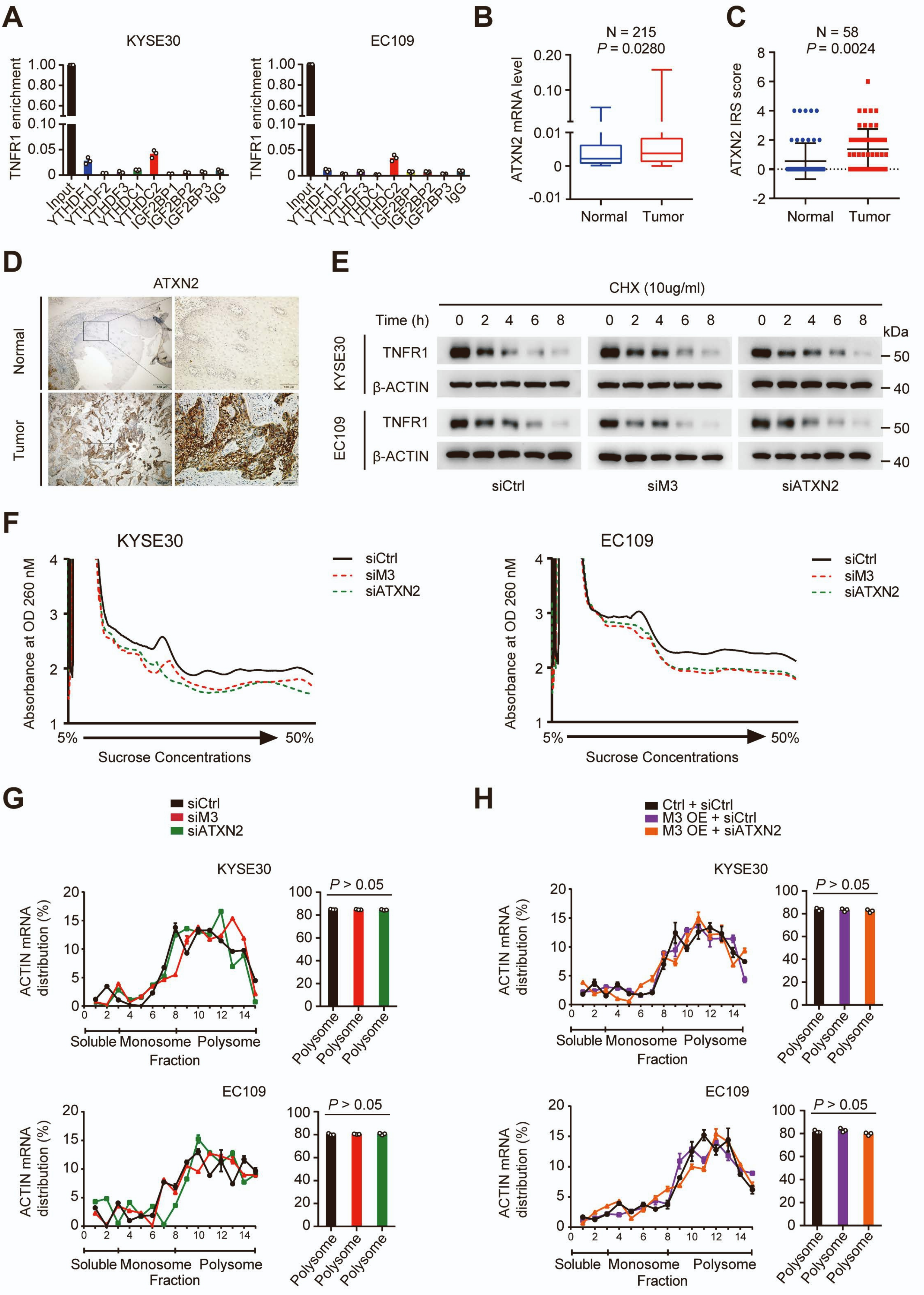

Figure S3. ATXN2 function as a TNFR1 m^{6}A mediator and promotes its translation in ESCC cells. Related to Figure 3.

(A) Association of common readers (YTHDF1/2/3, YTHDC1/2, IGF2BP1/2/3) with TNFR1 determined by RNA immunoprecipitation (RIP) assays. IgG served as the negative control. (B) mRNA levels of ATXN2 in ESCC samples compared with paired normal tissues (SYSUCC cohort, N = 215). (C, D) Quantification of IHC staining (C, SYSUCC cohort, N = 58) and representative IHC images of ATXN2 protein levels in ESCC tumors and paired adjacent normal tissues (D). Scale bar, 500 μm (left) and 100 μm (right). (E) ESCC cells with METTL3/ATXN2 knockdown and control cells were treated with cycloheximide (CHX; 10 μg/ml) for the indicated periods of time. TNFR1 levels were analyzed by immunoblotting. (F) The polysome profiling of ESCC cells with the indicated treatments. (G) Polysome fraction analysis in cells with the indicated treatments. The level of β-ACTIN mRNA in each gradient fraction was measured by qPCR and plotted as a percentage (G, H). The results of (A, G, H) are from at least 3 experiments, and data in (A, G, H) are mean ± SEM. *P*-values were calculated by two-sided Student's *t*-test (*$P < 0.05$, **$P < 0.01$ and ***$P < 0.001$) in (G, H). *P* values were calculated by two-sided paired Wilcoxon signed-rank test in (B) and (C).

Li et al_Supplementary Figure 4

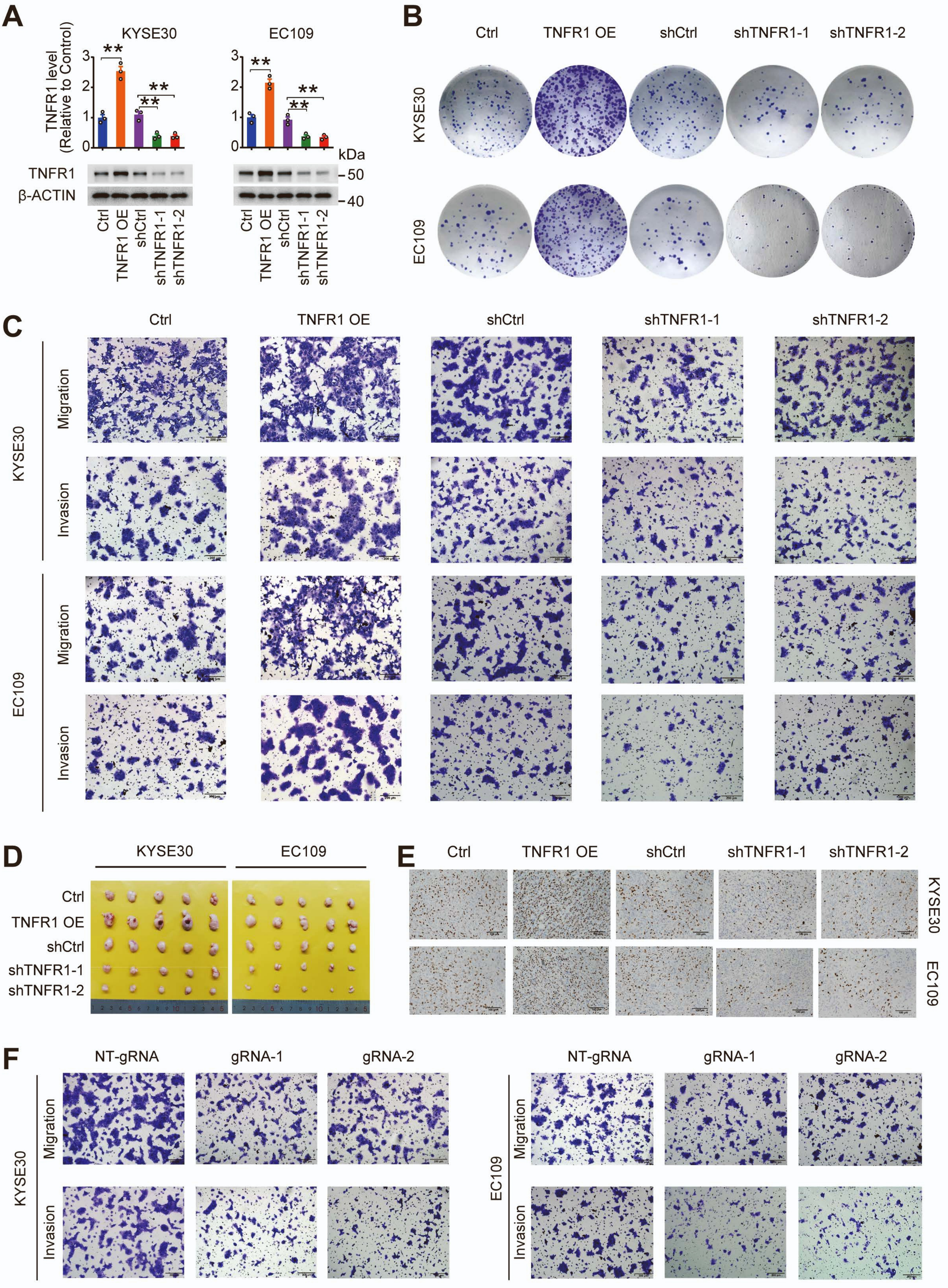

Figure S4. Effects of TNFR1 on ESCC cell malignant phenotypes. Related to Figure 5.

(A) Efficiency of TNFR1 overexpression and knockdown in ESCC cells. Data are the mean ± SEM from 3 independent experiments; **, $P < 0.01$, Student's t-test. (B) Colony formation assays were performed to detect the long-term proliferation capacity of KYSE30 and EC109 cells with TNFR1 overexpression or knockdown. (C) Representative images showing the effects of TNFR1 overexpression or knockdown on cell migration and invasion. Scale bars, 200 μm. (D) Effects of TNFR1 overexpression or knockdown on subcutaneous ESCC xenograft growth in mice. Images of xenograft tumors. (E) Representative IHC images of Ki67 levels in xenograft tumors from (D). Scale bars, 100 μm. (F) Representative pictures showing the effects on cell migration and invasion when removing TNFR1 m^6A modifications. Scale bars, 200 μm. The results of (A) are from at least 3 experiments, and the data of (D, E) are from 5 mice. Data in (A) are mean ± SEM. P values were calculated by two-sided Student's t-test (*$P < 0.05$, **$P < 0.01$ and ***$P < 0.001$) in (A).

Li et al_Supplementary Figure 5

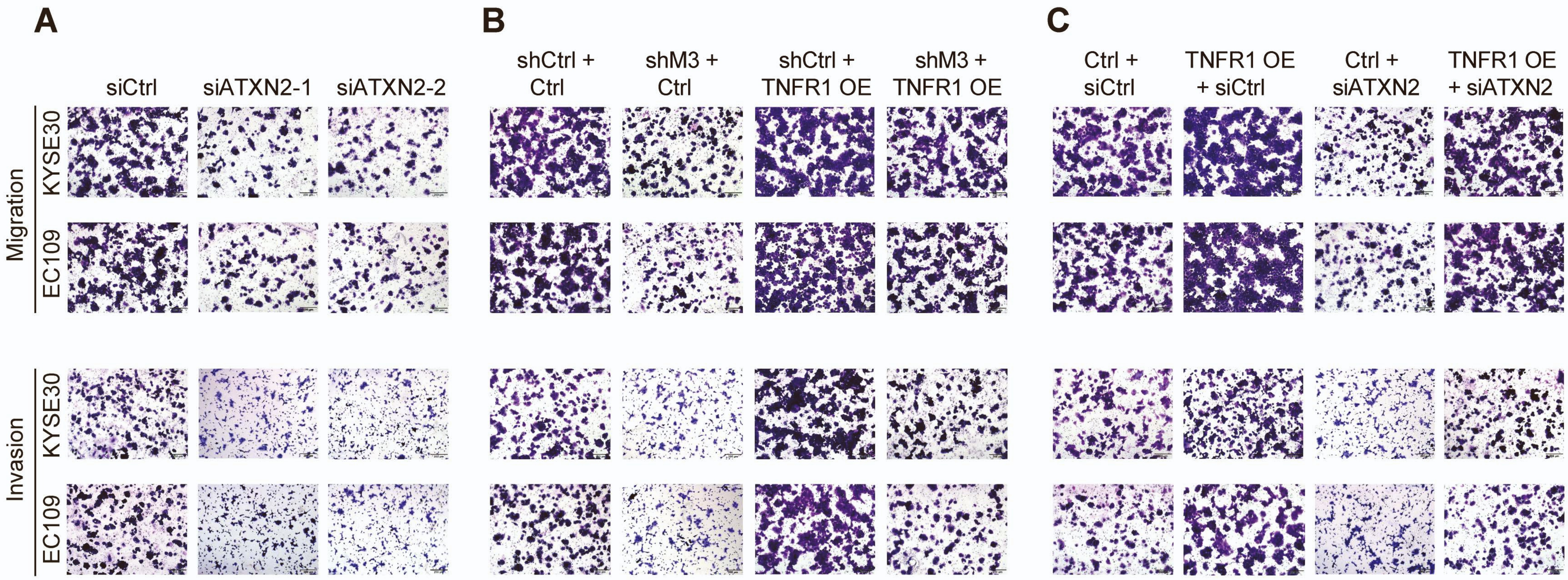

Figure S5. Effects of ATXN2 knockdown and rescue assays on ESCC malignant phenotypes and MAPK/NF-κB activities. Related to Figure 6.

(A) Representative images showing the effects of ATXN2 knockdown on cell migration and invasion. (B) Representative images showing the effects of TNFR1 overexpression on cell migration and invasion in cells with METTL3 knockdown (shM3). (C) Representative images showing the effects of ATXN2 depletion on cell migration and invasion in cells with TNFR1 overexpression (TNFR1 OE). Scale bars, 200 μm in (A−C).

Table S1. Genes involved in TNFR1 related pathways and their m^{6}A levels in ESCC.

Table S2. Baseline and clinical characteristics of patients with ESCC in this study.

**Variable**	**All cases (*N* = 215)**	**Alive (*N* = 93)**	**Deceased (*N* = 122)**	***P* value[d]**
Age, mean (S.E.M.[a])	60.09 (0.62)	59.86 (0.88)	60.27 (0.87)	0.744
Sex, *N* (%)				0.115
Male	166 (77.2)	67 (72.0)	99 (81.1)	
Female	49 (22.8)	26 (28.0)	23 (18.9)	
Family history, *N* (%)				0.631
Yes	52 (24.2)	21 (22.6)	31 (25.4)	
No	163 (75.8)	72 (77.4)	91 (74.6)	
Smoking status[b], *N* (%)				0.351
Ever	137 (63.7)	56 (60.2)	81 (66.4)	
Never	78 (36.3)	37 (39.8)	41 (33.6)	
Drinking status[b], *N* (%)				0.460
Ever	94 (43.7)	38 (40.9)	56 (45.9)	
Never	121 (56.3)	55 (59.1)	66 (54.1)	
Differentiation, *N* (%)				0.858
Well	37 (17.2)	17 (18.3)	20 (16.4)	
Moderate	114 (53.0)	50 (53.8)	64 (52.5)	
Poor	64 (29.8)	26 (27.9)	38 (31.1)	
Tumor stage[c], *N* (%)				< 0.0001
I	13 (6.0)	8 (8.6)	5 (4.1)	
II	93 (43.3)	54 (58.1)	39 (32.0)	
III	101 (47.0)	28 (30.1)	73 (59.8)	
IV	8 (3.7)	3 (3.2)	5 (4.1)	
Treatment, *N* (%)				0.283
Surgery Only	203 (94.4)	87 (93.5)	116 (95.1)	
Surgery + Chemotherapy	5 (2.3)	3 (3.2)	2 (1.6)	
Surgery + Radiotherapy	2 (1.0)	2 (2.2)	0 (0.0)	
Surgery + Chemoradiotherapy	5 (2.3)	1 (1.1)	4 (3.3)	

a) S.E.M., standard error of mean.

b) Patients were defined as nonsmokers who smoke an average of <1 cigarette/day and for <1 year during their lifetime; other people were defined as smokers. Patients were classified as drinkers who drink at least twice a week and continuously for at least 1 year in their lifetime; other people were defined as nondrinkers.

c) Tumor stage were defined according to the 7th edition of the AJCC Cancer Staging System.

d) *P* value was calculated by two-sided Chi-square test/Fisher's exact test.

Table S3. Baseline and clinical characteristics of patients with ESCC used for IHC in this study.

**Variable**	**All cases (*N* = 58)**	**Alive (*N* = 24)**	**Deceased (*N* = 34)**	***P* value[d]**
Age, mean (S.E.M.[a])	62.24 (1.14)	62.17 (1.44)	62.29 (1.68)	0.957
Sex, *N* (%)				0.313
Male	46 (79.3)	17 (70.8)	29 (85.3)	
Female	12 (20.7)	7 (29.2)	5 (14.7)	
Family history, *N* (%)				1.000
Yes	13 (22.4)	5 (20.8)	8 (23.5)	
No	45 (77.6)	19 (79.2)	26 (76.5)	
Smoking status[b], *N* (%)				0.352
Ever	39 (67.2)	14 (58.3)	25 (73.5)	
Never	19 (32.8)	10 (41.7)	9 (26.5)	
Drinking status[b], *N* (%)				1.000
Ever	26 (44.8)	11 (45.8)	15 (44.1)	
Never	32 (55.2)	13 (54.2)	19 (55.9)	
Differentiation, *N* (%)				0.934
Well	14 (24.1)	6 (25.0)	8 (23.5)	
Moderate	28 (48.3)	12 (50.0)	16 (47.1)	
Poor	16 (27.6)	6 (25.0)	10 (29.4)	
Tumor stage[c], *N* (%)				0.224
I	3 (5.2)	2 (8.3)	1 (2.9)	
II	22 (38.0)	12 (50.0)	10 (29.4)	
III	31 (53.4)	9 (37.5)	22 (64.8)	
IV	2 (3.4)	1 (4.2)	1 (2.9)	

a) S.E.M., standard error of mean.

b) Patients were defined as nonsmokers who smoke an average of <1 cigarette/day and for <1 year during their lifetime; other people were defined as smokers. Patients were classified as drinkers who drink at least twice a week and continuously for at least 1 year in their lifetime; other people were defined as nondrinkers.

c) Tumor stage were defined according to the 7th edition of the AJCC Cancer Staging System.

d) *P* value was calculated by two-sided Chi-square test/Fisher's exact test.

Table S4. Proteins identified in RNA pulldown and mass spectrometry analysis with TNFR1[m^{6}A] or TNFR1[A]

Table S5. Characteristics of ESCC patients for m^{6}A-seq in this study.

Sample ID	Gender	Age	Differentiation	Smoking status[a]	Drinking status[b]	Family history	Tumor stage[c]
320069	Male	53	Poor	Smoker	Nondrinker	No	III
318599	Male	53	Moderate	Smoker	Drinker	Yes	III
322954	Male	68	Moderate	Smoker	Drinker	No	III
320754	Male	59	Poor	Smoker	Nondrinker	No	III
349968	Male	61	Moderate	Smoker	Drinker	No	III
345487	Female	65	Poor	Nonsmoker	Nondrinker	No	III
350991	Male	62	Moderate	Nonsmoker	Drinker	Yes	II
335239	Male	53	Moderate	Smoker	Nondrinker	No	III

a) Patients were defined as nonsmokers who smoke an average of <1 cigarette/day and for <1 year during their lifetime; other people were defined as smokers.

b) Patients were classified as drinkers who drink at least twice a week and continuously for at least 1 year in their lifetime; other people were defined as nondrinkers.

c) Tumor stage were defined according to the 7th edition of the AJCC Cancer Staging System.

Table S6. Primers, oligonucleotides and sgRNAs used in this study.

**qRT-PCR**	Primer sequence (5' → 3')
METTL3-Forward	GTGACTATGGAACCAAGGAGGAG
METTL3-Reverse	TAAGGAAAGAGCAGTCACCTAAAGA
METTL14-Forward	TGCAGCACCTCGATCATTTATTT
METTL14-Reverse	AAGTCTTAGTCTTCCCAGGATTGTT
WTAP-Forward	GCCCAACTGAGATCAACAATGG
WTAP-Reverse	TGGCTATCAGGCGTAAACTTCC
VIRMA-Forward	TCCCAACGATGGCACGAAT
VIRMA-Reverse	TTGCAGCACACCAGGGTGAGC
TNFR1-Forward	AACGGTGGAAGTCCAAGCTCTAC
TNFR1-Reverse	AAGGTGGAACTGGGCACGGGA
ATXN2-Forward	CCTTCAATACTTAGTAACACGGAGCA
ATXN2-Reverse	CATTGGGATTCAATGTTGATTTCCTAA
β-ACTIN-Forward	ACAGAGCCTCGCCTTTGCCGAT
β-ACTIN-Reverse	CTTGCACATGCCGGAGCCGTT
**m^6A-RIP-qPCR**	Primer sequence (5' → 3')
TNFR1-Forward	TGCCTGGACAAGCACATAGCAAG
TNFR1-Reverse	GTGTATGTACAAAAGTCCACAGCTCC
**PAR-CLIP-qPCR**	Primer sequence (5' → 3')
TNFR1-Forward	TGCCTGGACAAGCACATAGCAAG
TNFR1-Reverse	GTGTATGTACAAAAGTCCACAGCTCC
**Select qPCR**	
TNFR1-select up oligonucleotides	tagccagtaccgtagtgcgtgTTGCTATGTGCTTG
TNFR1-select down oligonucleotides	5phos/CCAGGCAGAGGGCACAGGAGcagagg ctgagtcgctgcat
qPCR up primer	ATGCAGCGACTCAGCCTCTG
qPCR down primer	TAGCCAGTACCGTAGTGCGTG
**dCas13b-ALKBH5**	
sgRNA-1	AAAACAAAACAAAACAAAAACAAAAAA AACTGCTTATGCA
sgRNA-2	CTGTGAAAAAGGCTCAGGGACGAACCAG GGGCCCCCGAGC

Table S7. Sequences of shRNAs or siRNAs and probes used in this study**.**

**shRNA or siRNA**	**Sequence (5' → 3')**
shControl	TTCTCCGAACGTGTCACGT
shMETTL3-1	GGAGATCCTAGAGCTATTA
shMETTL3-2	GCACATCCTACTCTTGTAA
shTNFR1-1	GCCATGCAGGTTTCTTTCTAA
shTNFR1-2	CATTGGTTTAATGTATCGCTA
siControl	UUCUCCGAACGUGUCACGUTT
siATXN2-1	GCCCAUGCCAGUGAAUCAATT
siATXN2-2	CCAGCUUACUCCACGCAAUTT
siMETTL3	GGAGAUCCUAGAGCUAUUATT
**Probe for RNA pulldown and EMSA**	**Sequence (5' → 3')**
TNFR1[A]	ACUUGGCACUCCUGUGCCCUCUGCCUGGA[A]CAAGCACAUAGCAAGCUGAAC
TNFR1[m^{6}A]	ACUUGGCACUCCUGUGCCCUCUGCCUGGA[m^{6}A]CAAGCACAUAGCAAGCUGAAC

Table S8. Antibodies utilized in this study.

Antibody	Application	Source	Catalog number
Rabbit anti-METTL3	WB, IHC	Abcam	ab195352
Rabbit anti-TNFR1	WB	Cell Signaling Technology	3736
Rabbit anti-Ki67	IHC	ZSGB-BIO	ZA-0502
Rabbit anti-YTHDF1	RIP	Abcam	ab220162
Rabbit anti-YTHDF2	RIP	Abcam	ab220163
Rabbit anti-YTHDF3	RIP	Abcam	ab220161
Rabbit anti-YTHDC1	RIP	Cell Signaling Technology	81504
Rabbit anti-YTHDC2	RIP	Cell Signaling Technology	46324
Rabbit anti-IGF2BP1	RIP	Abcam	ab184305
Mouse anti-IGF2BP2	RIP	Abcam	ab128175
Rabbit anti-IGF2BP3	RIP	Abcam	ab177477
Rabbit anti-ATXN2	WB, RIP, CLIP	Proteintech	21776-1-AP
Rabbit anti-THRAP3	WB, RIP	Invitrogen	A300-956A
Rabbit anti-CCT5	WB, RIP	Invitrogen	PA5-22093
Rabbit anti-MTREX	WB, RIP	Abcam	ab70551
Rabbit anti-p65	WB	Cell Signaling Technology	8242
Rabbit anti-phospho p65	WB	Cell Signaling Technology	3033
Rabbit anti-ERK1/2	WB	Abcam	ab17942
Rabbit anti-phospho ERK1/2	WB	Abcam	ab76299
Rabbit anti-p38	WB	Cell Signaling Technology	8690
Rabbit anti-phospho p38	WB	Cell Signaling Technology	4511
Mouse anti-6-methyladenosine	RIP	Synaptic Systems	202003
Rabbit anti-ALKBH5	WB	Merckmillipore	ABE547
Mouse anti-ACTIN	WB	Proteintech	66009-1-Ig
